# Nonparametric Bayesian Adjustment of Unmeasured Confounders in Cox Proportional Hazards Models

**DOI:** 10.1002/sim.70360

**Published:** 2026-01-22

**Authors:** Shunichiro Orihara, Shonosuke Sugasawa, Tomohiro Ohigashi, Keita Hirano, Tomoyuki Nakagawa, Masataka Taguri

**Affiliations:** ^1^ Department of Health Data Science Tokyo Medical University Tokyo Japan; ^2^ Graduate School of Economics Keio University Tokyo Japan; ^3^ Department of Information and Computer Technology, Faculty of Engineering Tokyo University of Science Tokyo Japan; ^4^ Department of Human Health Sciences, Graduate School of Medicine Kyoto University Kyoto Japan; ^5^ School of Data Science Meisei University Tokyo Japan

**Keywords:** clustering, Dirichlet process mixture, general Bayes, time‐to‐event, UK Biobank

## Abstract

Unmeasured confounders pose a major challenge in accurately estimating causal effects in observational studies. To address this issue when estimating hazard ratios (HRs) using Cox proportional hazards models, several methods, including instrumental variables (IVs) approaches, have been proposed. However, these methods often face limitations, such as weak IV problems and restrictive assumptions regarding unmeasured confounder distributions. In this study, we introduce a novel nonparametric Bayesian procedure that provides accurate HR estimates while addressing these limitations. A key assumption of our approach is that unmeasured confounders exhibit a cluster structure. Under this assumption, we integrate two remarkable Bayesian techniques, the Dirichlet process mixture (DPM) and general Bayes (GB), to simultaneously (1) detect latent clusters based on the likelihood of exposure and outcome variables and (2) estimate HRs using the likelihood constructed within each cluster. Notably, leveraging DPM, our procedure eliminates the need for IVs by identifying unmeasured confounders under an alternative condition. Additionally, GB techniques remove the need for explicit modeling of the baseline hazard function, distinguishing our procedure from traditional Bayesian approaches. Simulation experiments demonstrate that the proposed Bayesian procedure outperforms existing methods in some performance metrics. Moreover, it achieves statistical efficiency comparable to the efficient estimator while accurately identifying cluster structures. These features highlight its ability to overcome challenges associated with traditional IV approaches for time‐to‐event data.

## 1 Introduction

In observational studies, the problem of unmeasured confounders, where certain confounders are unobserved or unknown, can lead to biased causal effect estimates. In such cases, the instrumental variable (IV) method is a commonly used approach for addressing this issue. In biometrics and related fields, several theoretical developments and practical applications of IV methods have emerged in recent years [1, 2]. Mendelian randomization (MR), which typically uses single nucleotide polymorphisms (SNPs) as IVs, is a notable example.

Time‐to‐event outcomes are common in biometrics, including in MR contexts [3, 4], and are often analyzed using the Cox Proportional Hazards Model (CPHM) [5], which summarizes causal effects as hazard ratios (HRs). Recently, several IV methods applicable to the CPHM have been proposed. Kianian et al. [6], Wang et al. [7], and Cui et al. [8] introduced weighing‐based estimators for marginal HRs, which can be interpreted as causal HRs [9]. While these approaches provide attractive interpretations of HRs as summaries of potential outcomes, they are limited to cases where the IV is a single, binary variable. If the IV is not binary, artificial dichotomization becomes necessary [10].

Building on the two‐stage residual inclusion (2SRI) approach, a commonly used IV method for binary outcomes [11], Martínez‐Camblor et al. [12] proposed a frailty model‐based HR estimator [13]. Although their method demonstrated useful properties under continuous exposure conditions, 2SRI may yield biased or unstable causal effect estimates in certain scenarios [14, 15, 16], particularly in weak IV situations [17].

Basu et al. [14] and Orihara et al. [16] demonstrated that full‐likelihood approaches generally produce more accurate estimates than 2SRI. The limited information maximum likelihood (LIML) estimator, which can be considered an IV method [18], employs a full‐likelihood framework and is known for its robustness in weak IV scenarios [19, 20, 21]. By extending this framework, Orihara et al. [21] proposed a LIML‐based HR estimator, however, their method relies on strong assumptions, including a common normal distribution for unmeasured confounders across subjects and the prior specification of a variance parameter.

In this study, we introduce a novel nonparametric Bayesian procedure that provides accurate HR estimates while addressing these limitations. While our approach shares foundational elements with LIML‐based methods, both of which use likelihood, it overcomes key limitations by relaxing distributional assumptions about unmeasured confounders. A key assumption of our approach is that unmeasured confounders exhibit a cluster structure. Under this assumption, we integrate two remarkable Bayesian techniques, the Dirichlet process mixture (DPM) [22, 23, 24] and general Bayes (GB) [25], to simultaneously (1) detect latent clusters based on the likelihood of exposure and outcome variables and (2) estimate HRs using the likelihood constructed within each cluster. Notably, leveraging DPM, our procedure eliminates the need for IVs by identifying unmeasured confounders under an alternative condition. This feature is similar to the results of Xu et al. [26], which treat unmeasured confounders as random effects. Additionally, when estimating HRs using the derived posterior distribution, GB techniques remove the need for explicit modeling of the baseline hazard function, distinguishing our procedure from traditional Bayesian approaches. While Bayesian methods have been applied in related contexts [27], our contribution is unique in applying general Bayes techniques to the CPHM while accommodating unmeasured confounders.

The remainder of this article is organized as follows. Section 2 describes the models and assumptions underlying our approach, with a particular focus on the cluster structure of unmeasured confounders. Additionally, we discuss the role of variables that can be considered IVs in our proposed procedure, in conjunction with exclusion restriction violations. Section 3 outlines the proposed Bayesian procedure along with its sampling algorithm. Section 4 evaluates the performance of the proposed procedure in comparison to previous approaches through simulation studies. Section 5 demonstrates the application of the proposed procedure using UK Biobank datasets.

## 2 Preliminaries

### 2.1 Cox Proportional Hazards Models With Cluster Structures

For i=1,…,n, let $T_{i}$ represent the survival time, $\delta_{i}$ be the censoring indicator (whether an event is observed, $\delta_{i}=1$, or not, $\delta_{i}=0$), and $A_{i}$ be the exposure variable. As auxiliary information, let $x_{i}$ represent the vector of covariates. Additionally, let $\lambda_{i}(t)$ denote the hazard function for subject i. We then consider a continuous exposure model and a CPHM for the outcome, described as

(2.1) $$\begin{array}{ll} A_{i} & =\alpha_{0i}+x_{i}^{⊤}\alpha_{xi}+\varepsilon_{i}, \end{array}$$

(2.2) $$\begin{array}{ll} \lambda_{i}(t) & =\lambda_{0i}(t)\exp\left({\tilde{x}}_{i}^{⊤}\beta \right), \end{array}$$

where $\varepsilon_{i}∼N(0,\sigma_{i}^{2})$ is an error term, and $\lambda_{0i}(t)$ is the subject‐specific baseline hazard function. Here, $\tilde{x}$ represents the vector of explanatory variables for the outcome model, including a (an observed value of A) and x; specifically, ${\tilde{x}}^{⊤}=\left(a,x^{⊤}\right)$ and $\beta^{⊤}=\left(\beta_{a},\beta_{x}^{⊤}\right)$. In this setting, we are particularly interested in the treatment effect $\beta_{a}$. Note that more complex model structures can be considered for both models; for instance, an interaction term between the exposure and confounders can be included in model (2.2).

From the models (2.1) and (2.2), the covariates $x_{i}$ are regarded as measured confounders. In standard causal inference contexts, the causal effect of interest can be estimated by adjusting for these measured confounders [9]. However, in our article, we consider a situation in which unmeasured confounders exist, arising from subject‐specific parameters. This point is discussed in the following sections.

The models (2.1) and (2.2) contain subject‐specific parameters, $\alpha_{0i}$, $\alpha_{xi}$, $\sigma_{i}^{2}$, and $\lambda_{0i}(t)$, which cannot be identified without structural assumptions. To address the high dimensionality of these parameters, we introduce a nonparametric Bayesian approach. Specifically, we assume that

(2.3) $$(\alpha_{0i},\alpha_{xi},\sigma_{i}^{2},\lambda_{0i})|P∼P,\;P∼\text{DP}(G_{0},\gamma ),\;i=1,\ldots ,n,$$

where P is a discrete random probability measure, and $\text{DP}(G_{0},\gamma )$ represents the Dirichlet process prior [22, 23, 24] for P, with base measure $G_{0}$ and precision parameter γ. When the data exhibit cluster structures, the Dirichlet process prior allows for the estimation of parameters with clustering, without requiring prior knowledge of the number or sizes of the clusters. The size of each cluster is influenced by the precision parameter γ.

Suppose we have n items {1,2,…,n} independently sampled from $\text{DP}(G_{0},\gamma )$, which are partitioned into $K_{n}$ distinct groups $\left(C_{1},\ldots ,C_{K_{n}}\right)$, where $N_{k}$ represents the size of the kth cluster for $k=1,\ldots ,K_{n}$. Let $s_{1},\ldots ,s_{n}\in \{1,\ldots ,K_{n}\}$ be the latent assignments for each subject. Then, the joint probability of $S=(s_{1},\ldots ,s_{n})$ is given by

(2.4) $$P(s_{1},\ldots ,s_{n};\gamma )=\overset{n}{\underset{i=1}{\prod }}p(s_{i}|s_{1},\ldots ,s_{i-1};\gamma ),$$

where the conditional probabilities follow the well‐known Chinese restaurant process:

(2.5) $$\begin{array}{cc} & p(s_{i}=k|s_{1},\ldots ,s_{i-1};\gamma )=\frac{N_{k}(i-1)}{i-1+\gamma },\;k=1,\ldots ,K_{i-1},\\ & p(s_{i}=K_{i-1}+1|s_{1},\ldots ,s_{i-1};\gamma )=\frac{\gamma }{i-1+\gamma }, \end{array}$$

where $N_{k}(i-1)=\sum_{j=1}^{i-1}I(s_{j}=k)$ is the size of the kth cluster induced by $s_{1},\ldots ,s_{i-1}$, and I(·) is the indicator function. Note that in our proposed method, the effects of unmeasured confounders can be interpreted as cluster‐specific parameters denoted as: $u_{i}:=(\alpha_{0s_{i}},\alpha_{xs_{i}},\sigma_{s_{i}}^{2},\lambda_{0s_{i}})$. This point is discussed in more detail in the next section.

### 2.2 Confounding Bias as Cluster Effect

As described in the previous section, the use of the Dirichlet process introduces latent partition structures for the n subjects. For the kth cluster, models (2.1) and (2.2) can be expressed as

$$A_{i}∼N(\alpha_{0k}+x_{i}^{⊤}\alpha_{xk},\sigma_{k}^{2}),\;\lambda_{i}(t)=\lambda_{0k}(t)\exp\left(a_{i}\beta_{a}+x_{i}^{⊤}\beta_{x}\right).$$

Thus, each cluster has its own intercept $\alpha_{0k}$, coefficients $\alpha_{xk}$, variance $\sigma_{k}^{2}$, and baseline hazard $\lambda_{0k}(t)$, which can be interpreted as a cluster effect resulting from unmeasured confounders.

**FIGURE 1:**
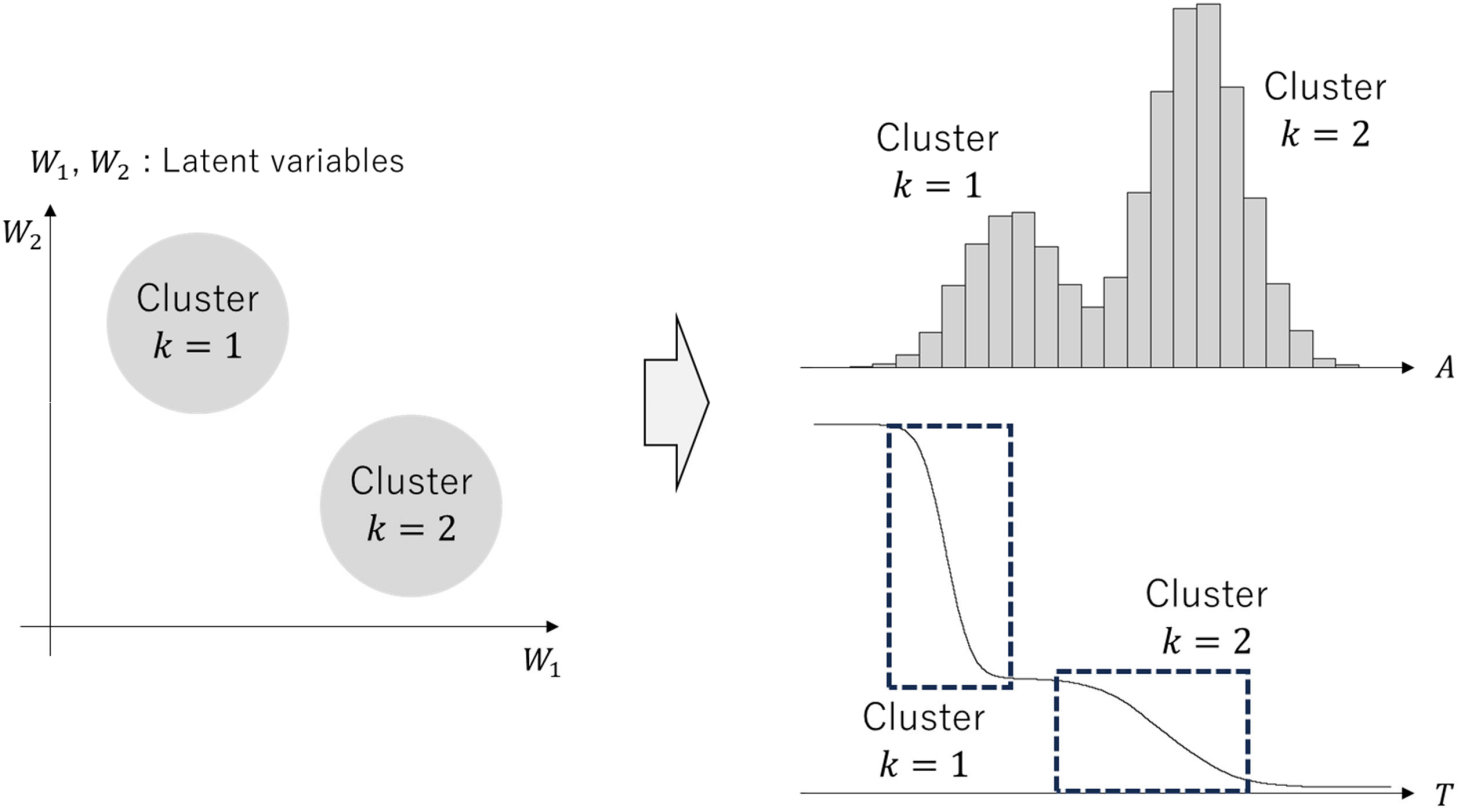
Concept of the impact of cluster effects on confounding bias (There are two clusters: one consists of subjects with small values of $W_{1}$ and large value of $W_{2}$, while the other consists of subjects with large values of $W_{1}$ and small value of $W_{2}$. Regarding the observed exposure variable A and the outcome variable T, subjects with larger values of $\alpha_{k}$ tend to have higher values of $\lambda_{0k}(t)$ in the later time periods).

For instance, consider a scenario where there are two clusters, k∈{1,2} (see Figure 1). In this example, there are two clusters: one consists of subjects with small values of $W_{1}$ and large value of $W_{2}$, while the other consists of subjects with large values of $W_{1}$ and small value of $W_{2}$, where $W_{1}$ and $W_{2}$ are some latent variables. Regarding the observed exposure variable A and the outcome variable T, subjects with larger values of $\alpha_{0k}$ tend to have higher values of $\lambda_{0k}(t)$ in the later time periods. Consequently, subjects with smaller/larger exposure values are more likely to experience the event of interest earlier/later. This suggests that the treatment effect $\beta_{a}$ could be biased if the cluster effects are not taken into account, specifically by ignoring the effects of $u_{i}$. This type of bias is a well‐known phenomenon referred to as “confounding bias.”

In this context, adjusting for unmeasured confounder effects is equivalent to conducting analyses that account for the cluster effect. In other words, to accurately estimate the treatment effect $\beta_{a}$, it is essential to consider the cluster effect. This setting implicitly assumes that unmeasured confounders have discrete supports, as described by Miao et al. [28] and Shi et al. [29]. Similarly, in the context of population stratification, unmeasured confounders can manifest as cluster‐based genetic ancestries, leading to confounding issues [30].

### 2.3 Importance of Common Exposure Effects Across Clusters

In models (2.1) and (2.2), IVs are not explicitly shown. In fact, (valid) IVs are not strictly required to construct our proposed estimation procedure; this is a key difference from previous IV methods. The key consideration for our proposed procedure is that the accuracy of the estimation relies on the plausibility of the likelihoods in both exposure and outcome models, similar to the assumption in Xu et al. [26]. Therefore, the fundamental assumptions differ from those of IV methods.

Here, suppose that the confounders and related coefficients can be decomposed into two parts: $X^{⊤}=(Z^{⊤},V^{⊤})$ and $\alpha_{xk}^{⊤}=(\alpha_{z}^{⊤},\alpha_{vk}^{⊤})$. Note that Z has a common effect $\alpha_{z}$ (≠0) on the exposure variable across clusters ($\alpha_{zk}\equiv \alpha_{z}$). Under these assumptions, Z is a common exposure effects across clusters (“common predictor” hereinafter) and plays an important role in accurately identifying each cluster, while V serves as a predictor within each cluster.

### 2.4 Comparison With Ordinary IV Methods

Z can be considered as an IV. In ordinary IV methods such as two‐stage least square (2SLS) or 2SRI, a valid IV must satisfy three conditions [19]:

1. The IV is associated with the treatment (relevance).
2. The IV affects the outcome only through its effect on the treatment (exclusion restriction).
3. The IV is independent of unmeasured confounders.

These assumptions enable the IV to serve as a surrogate for “random allocation,” which in turn allows for the estimation of a valid causal effect.

In 2SLS, the first stage involves regressing the treatment variable on the IVs. For instance, we consider the following model:

$$E[A|Z_{i}]=\alpha_{0}+Z_{i}^{⊤}\alpha_{z}.$$

In the second stage, the predicted treatment values obtained from the first stage, denoted as ${Â}_{i}:={\hat{\alpha }}_{0}+Z_{i}^{⊤}{\hat{\alpha }}_{z}$, are used as regressors in a model for the outcome variable. Specifically, we consider the following model:

$$\lambda_{i}^{2SLS}(t)=\lambda_{0}(t)\exp\left({Â}_{i}\beta_{a}+x_{i}^{⊤}\beta_{x}\right).$$

These predicted values are functions of the IVs and therefore do not contain the effects of unmeasured confounders. In 2SRI, the first stage is the same as in 2SLS. However, in the second stage, both the treatment value and the residuals from the first‐stage regression are included as regressors in the outcome model. These residuals, denoted as ${\hat{r}}_{i}:=A_{i}-{Â}_{i}$, are interpreted as proxies for unmeasured confounders. By incorporating the residuals, the following outcome model is considered:

$$\lambda_{i}^{2SRI}(t)=\lambda_{0}(t)\exp\left(a_{i}\beta_{a}+x_{i}^{⊤}\beta_{x}+{\hat{r}}_{i}\beta_{r}\right).$$

Note that this estimation strategy is similar to Heckman's two‐stage estimation procedure [31], and such approaches can accommodate misspecification of the treatment model [2].

In contrast, in our proposed model, Z plays a different role. The core assumption is that the model structures are valid for the dataset as discussed in Section 2.2 and 2.3; the identification condition is fundamentally different from that of IV methods. Importantly, Z is not necessarily excluded from the outcome model (2.2), which represents a violation of the exclusion restriction. Therefore, Z does not necessarily need to be a valid IV; allowing for the possibility of an invalid IV due to horizontal pleiotropy, as discussed in MR contexts [32], may be reasonable. In short, the role of Z in our proposed procedure differs from its role in ordinary IV methods; while it enhances statistical efficiency in our method, it relates to the validity of causal effect estimates in ordinary IV methods.

## 3 Nonparametric Bayesian Adjustment

To estimate the target parameter $\beta ={\left(\beta_{a},\beta_{x}^{⊤}\right)}^{⊤}={\left(\beta_{a},\beta_{z}^{⊤},\beta_{v}^{⊤}\right)}^{⊤}$ in the presence of unmeasured confounders, we propose a novel nonparametric Bayesian estimation method along with its sampling algorithm. For the following discussions, we assume that the censoring time, for events other than the event of interest, is independent of the event time $t_{i}$ across clusters k, given A and X. This assumption is similar to that made by Orihara et al. [21].

### 3.1 Posterior for Nonparametric Bayesian Adjustment

Given the cluster assignment, S, the outcome model in (2.2) reduces to the following clustered CPHM:

$$\lambda_{i}(t)|(s_{i}=k)=\lambda_{0k}(t)\exp\left(a_{i}\beta_{a}+x_{i}^{⊤}\beta_{x}\right),\;k=1,\ldots ,K_{n}.$$

To make inference on β, we consider the clustered partial likelihood, $\prod_{k=1}^{K_{n}}\prod_{i\in C_{k}}\ell_{ik}(\beta )$, where

(3.1) $$\begin{array}{l} \ell_{ik}(\beta ):={\left\{\frac{\exp(a_{i}\beta_{a}+x_{i}^{⊤}\beta_{x})}{\sum_{\ell \in R_{k}(T_{i})}\exp(a_{\ell }\beta_{a}+x_{\ell }^{⊤}\beta_{x})}\right\}}^{\delta_{i}}, \end{array}$$

and $R_{k}(t)$ denotes the cluster‐wise risk set at time t. A notable feature of the partial likelihood (3.1) is that it is free from the nuisance baseline hazard function, $\lambda_{0k}(\cdot )$, allowing us to focus on the posterior distribution of β. While traditional Bayesian inference for the CPHM typically requires modeling the baseline hazard function, such as using a Gamma process prior [33, 34, 35], the framework known as the “general posterior” [25] allows us to define an analog of a posterior distribution based on synthetic likelihoods, such as (3.1).

The exposure model in (2.1), given the latent partition, can be expressed as

(3.2) $$\begin{array}{cc} & A_{i}|(s_{i}=k)∼N(\alpha_{0k}+z_{i}^{⊤}\alpha_{z}+v_{i}^{⊤}\alpha_{vk},\sigma_{k}^{2}),\;k=1,\ldots ,K_{n}. \end{array}$$

This model is similar to, but not identical to, the likelihood assumed in Orihara et al. [21], which assumes that all subjects share the same distribution ($K_{n}\equiv 1$), and σ is a fixed value.

Let $D_{i}=(T_{i},\delta_{i},A_{i},x_{i})$ and $D=(D_{1},\ldots ,D_{n})$ be the set of observations. Then, the joint (general) posterior can be obtained as

(3.3) $$\begin{array}{cc} & p(\beta ,\alpha_{0},\alpha_{v},\alpha_{z},\Sigma ,\gamma ,s|D)\\ & \;\propto \pi (\beta ,\alpha_{z},\gamma )\pi_{G_{0}}(\alpha_{0},\alpha_{v},\Sigma )P(s_{1},\ldots ,s_{n};\gamma )\\ & \;\times \overset{K_{n}}{\underset{k=1}{\prod }}\underset{i\in C_{k}}{\prod }\ell_{ik}(\beta )\phi \left(A_{i};\alpha_{0k}+z_{i}^{⊤}\alpha_{z}+v_{i}^{⊤}\alpha_{vk},\sigma_{k}^{2}\right), \end{array}$$

where $\pi (\beta ,\alpha_{z},\Sigma ,\gamma )$ is a prior distribution on (global) parameters and $\pi_{G_{0}}(\alpha_{0},\alpha_{v},\Sigma )$ is a prior distribution on cluster‐wise parameters generated from the baseline distribution $G_{0}$. Here ϕ(x;a,b) denotes the density function of the normal distribution with mean a and variance b. Additionally, $P(s_{1},\ldots ,s_{n};\gamma )$ and $\ell_{ik}(\beta )$ are defined in (2.4) and (3.1), respectively. Note that the likelihood for the posterior (3.3) is fundamentally composed of

(3.4) $$\begin{array}{l} f(T_{i},A_{i}|X_{i},s_{i}=k)=f(T_{i}|A_{i},X_{i},s_{i}=k)\times f(A_{i}|X_{i},s_{i}=k); \end{array}$$

this likelihood decomposition is natural in causal inference contexts [36].

### 3.2 Posterior Computation

In this section, we assume the prior distributions, $\beta_{a}∼N(m_{\beta_{a}},\tau_{\beta_{a}}^{2})$, $\beta_{v}∼N(m_{\beta_{v}},\Sigma_{\beta_{v}})$, $\alpha_{vk}∼N(m_{\alpha_{vk}},\Sigma_{\alpha_{vk}})$, $\alpha_{z}∼N(m_{\alpha_{z}},\Sigma_{\alpha_{z}})$, $\alpha_{0k}∼N(m_{\alpha_{0k}},\tau_{\alpha_{0k}}^{2})$, $\sigma_{k}^{2}∼\text{IG}(a_{\sigma k},b_{\sigma k})$, and $\gamma ∼\text{Ga}(a_{\gamma },b_{\gamma })$ as default priors. For $\beta_{z}$, a shrinkage prior is applied; for more details, see Appendix A. Here, all prior distributions are assumed to be mutually independent.

Based on the joint posterior (3.3), the full conditional distributions of each parameter and latent assignment are obtained as follows:

– (Sampling of $\alpha_{z}$) The full conditional distribution of $\alpha_{z}$ is proportional to
$$\pi (\alpha_{z})\overset{K_{n}}{\underset{k=1}{\prod }}\underset{i\in C_{k}}{\prod }\phi \left(A_{i};\alpha_{0k}+z_{i}^{⊤}\alpha_{z}+v_{i}^{⊤}\alpha_{vk},\sigma_{k}^{2}\right).$$
– (Sampling of $\alpha_{0}$ and $\alpha_{v}$) For $k=1,\ldots ,K_{n}$, the full conditional distribution of $\left(\alpha_{0k},\alpha_{vk}\right)$ is proportional to
$$\pi (\alpha_{0k},\alpha_{vk})\underset{i\in C_{k}}{\prod }\phi \left(A_{i};\alpha_{0k}+z_{i}^{⊤}\alpha_{z}+v_{i}^{⊤}\alpha_{vk},\sigma_{k}^{2}\right).$$
– (Sampling of Σ) For $k=1,\ldots ,K_{n}$, the full conditional distribution of $\sigma_{k}^{2}$ is proportional to
$$\pi (\sigma_{k}^{2})\underset{i\in C_{k}}{\prod }\phi \left(A_{i};\alpha_{0k}+z_{i}^{⊤}\alpha_{z}+v_{i}^{⊤}\alpha_{vk},\sigma_{k}^{2}\right).$$

Since the likelihood for the exposure variable is assumed to follow a normal distribution, with normal priors for the mean structure and an inverse‐Gamma prior for the variance, posterior sampling can be implemented straightforwardly.

When interested in a binary treatment, we construct a similar posterior sampling algorithm by modifying the likelihood, for example, to a logistic regression model.

– (Sampling of S) For i=1,…,n, the full conditional distribution of $s_{i}\in \{1,\ldots ,K_{n}\}$, namely, full conditional probability being $s_{i}=k$ is proportional to
$$P(s_{1},\ldots ,s_{n};\gamma )\ell_{ik}(\beta_{a},\beta_{x})\phi \left(A_{i};\alpha_{0k}+z_{i}^{⊤}\alpha_{z}+v_{i}^{⊤}\alpha_{vk},\sigma_{k}^{2}\right)$$
  for $k=1,\ldots ,K_{n}$.
– (Sampling of γ) The full conditional distribution of γ is proportional to
$$\pi (\gamma )P(s_{1},\ldots ,s_{n};\gamma ).$$

In the sampling step for S and γ, the Chinese restaurant process (2.5) will be used in the following simulation and real data experiments. Detailed sampling algorithms for sampling of S and γ are described in Appendix A.

– (Sampling of $\beta_{a}$ and $\beta_{x}$) For $k=1,\ldots ,K_{n}$, the full conditional distribution of $\left(\beta_{a},\beta_{x}\right)$ is proportional to
$$\pi (\beta_{a},\beta_{x})\overset{K_{n}}{\underset{k=1}{\prod }}\underset{i\in C_{k}}{\prod }\ell_{ik}(\beta_{a},\beta_{x}),$$
  where $\ell_{ik}(\beta_{a},\beta_{x})$ is given in (3.1). Note that the cumulative hazard function can be estimated using the Breslow estimator with the posterior samples of β [37]; however, the Bayesian justification of this plug‐in estimator remains unclear.

We use Stan to generate random samples of β from the posterior distribution of the outcome model parameters.

Remark 1 Using information from the treatment effect model

To estimate β, the treatment model does not necessarily need to be included in the above sampling step. However, since the decomposition (3.4) holds only within each stratum in our proposed model, cluster‐shared parameters such as β may benefit from the information provided by the treatment model. This information sharing may help improve the estimation efficiency of β; see Appendix B.5.5 for further discussion. This shares some conceptual similarities to Hahn et al. [38].

## 4 Simulation Experiments

In this section, we evaluate the performance of our proposed methods in comparison to several competitors, which are explained later, using simulation data examples. The number of iterations for all simulation examples was set to 200. For more details about the simulation experiments, see Appendix B.

### 4.1 Data‐Generating Mechanism

First, we describe the Data‐generating Mechanism (DGM) used in this simulation. We consider a cluster‐labeled variable, denoted as $K_{i}\overset{i.i.d.}{∼}Multi\left(1,\left(1/2,1/3,1/6\right)\right)$. This variable, labeled as K=0, K=1, and K=2, potentially introduces confounding bias. Additionally, we consider one common predictor shared across clusters and one measured confounder, denoted as $Z_{i}\overset{i.i.d.}{∼}Gamma(2,2)$ and $V_{i}\overset{i.i.d.}{∼}Ber(0.5)$, respectively. Using these variables, the exposure variable is defined as $A|z_{i},v_{i},k∼N\left(\alpha_{0k}+z_{i}\alpha_{z}+v_{i}\alpha_{vk},0.5^{2}\right)$.

In our simulation experiments, we consider two main settings: “Easy to identify” and “Hard to identify.” In the former setting, the cluster structure can be identified intuitively, whereas it is challenging to identify in the latter. Within these two main settings, we examine four scenarios: (a) strong common predictor and strong confounder, (b) weak common predictor and strong confounder, (c) strong common predictor and weak confounder, and (d) weak common predictor and weak confounder. All settings are summarized in Table B.1 in Appendix B.

The outcome model has a somewhat complex DGM; in any case, the distribution includes three distinct clusters. For more details, see Appendix B. Censoring occurs in approximately 10%–15% of cases, derived from an exponential distribution. Crude analyses of a simulation dataset are summarized in Figures B.1 to B.4 in Appendix B.

### 4.2 Estimating Methods and Performance Metrics

We considered five methods for estimating the HR. As discussed in Section 2.4, several IV methods were also considered since the common predictor Z can be regarded as a valid IV. Specifically, we evaluated the proposed method (“Proposed”), two reference methods with and without unmeasured confounder information (“Infeasible” and “Naive”), and two reference IV methods (“2SLS” and “2SRI”).

We evaluated the various methods based on mean, bias, empirical standard error (ESE), root mean squared error (RMSE), coverage probability (CP), and boxplot of estimated parameters from 200 iterations. In our proposed method, a point estimate is the posterior mean. The bias and RMSE were calculated as follows: $bias={\bar{\hat{\beta }}}_{a}-\beta_{a}^{0}$, $RMSE=\sqrt{\frac{1}{200}\sum_{k=1}^{200}{\left({\hat{\beta }}_{ak}-\beta_{a}^{0}\right)}^{2}}$, where ${\bar{\hat{\beta }}}_{a}=\frac{1}{200}\sum_{k=1}^{200}{\hat{\beta }}_{ak}$, ${\hat{\beta }}_{ak}$ is the estimate of each method and iteration, and $\beta_{a}^{0}$ (=−0.1) is the true value of the log–hazard ratio. The CP refers to the proportion of cases where the confidence interval or credible interval includes $\beta_{a}^{0}$. The boxplot summarize estimated HR, where the true HR is $\exp\{\beta_{a}^{0}\}$ (=exp{−0.1}≈0.905).

### 4.3 Simulation Results

**FIGURE 2:**
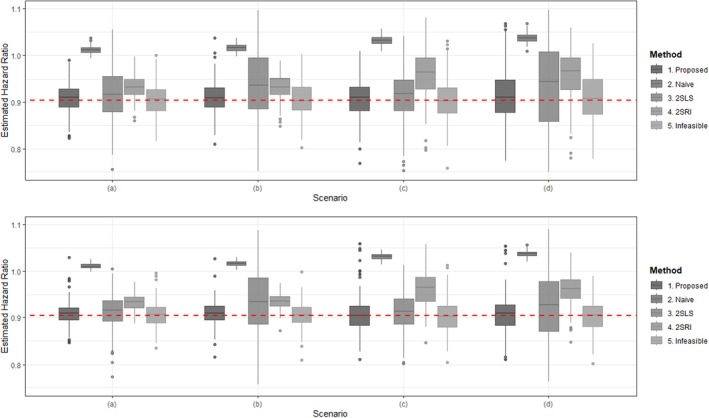
Box plots of hazard ratio estimates for each method in “Easy to identify” setting: The iteration time is 200. The true values of hazard ratio is exp{−0.1}≈0.905. (Upper figure: the sample is 600; lower figure: the sample is 1200; (a) strong common predictor and strong confounder; (b) weak common predictor and strong confounder; (c) strong common predictor and weak confounder; and (d) weak common predictor and weak confounder.)

**FIGURE 3:**
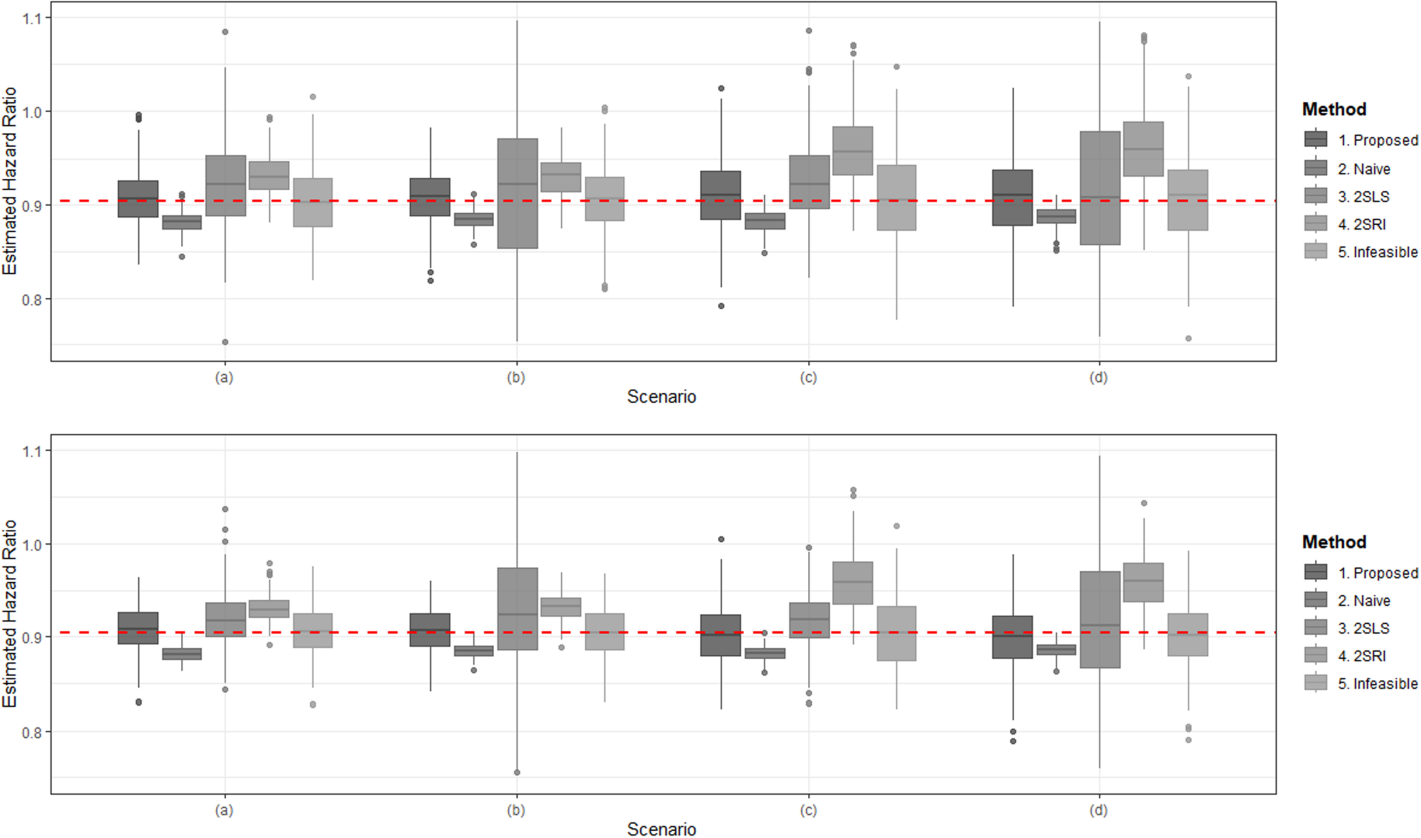
Box plots of hazard ratio estimates for each method in “Hard to identify” setting: The iteration time is 200. The true values of hazard ratio is exp{−0.1}≈0.905 (red dashed line) (Upper figure: the sample is 600; lower figure: the sample is 1200; (a) strong common predictor and strong confounder; (b) weak common predictor and strong confounder; (c) strong common predictor and weak confounder; and (d) weak common predictor and weak confounder).

All settings are summarized in Table 1 and Figures 2 and 3. Our proposed method produces results that are relatively similar to those of the infeasible method and can identify the cluster structure without any prior cluster information. In contrast, as expected, the naive method exhibits an obvious bias. Under strong common predictor (i.e., IV) scenarios, 2SLS is somewhat efficient, but it shows large variance in weak common predictor scenarios, which is a well‐known result. 2SRI provides more stable results compared to 2SLS but still displays clear bias. This bias likely arises from the residuals' inability to accurately capture the distribution of unmeasured confounders [15, 21].

**TABLE 1:** Summary of Hazard Ratio Estimates: The iteration time is 200, and the true values of the log hazard ratio is −0.1.

			log–hazard ratio							
			n=600				n=1200			
Setting	Scenario	Method	Bias	ESE	RMSE	CP	Bias	ESE	RMSE	CP
Easy to identify	(a)	1. Proposed	0.004	0.034	0.034	0.985	0.004	0.028	0.028	0.975
		2. Naive	0.111	0.008	0.112	0.000	0.111	0.006	0.111	0.000
		3. 2SLS	−0.014	0.266	0.267	0.855	0.007	0.042	0.043	0.875
		4. 2SRI	0.031	0.026	0.040	0.680	0.031	0.017	0.035	0.505
		5. Infeasible	0.000	0.040	0.040	0.955	0.001	0.029	0.029	0.945
	(b)	1. Proposed	0.005	0.037	0.037	0.975	0.005	0.029	0.029	0.970
		2. Naive	0.116	0.007	0.116	0.000	0.116	0.005	0.116	0.000
		3. 2SLS	−0.548	6.477	6.500	0.835	0.055	1.292	1.293	0.880
		4. 2SRI	0.029	0.028	0.041	0.650	0.032	0.016	0.036	0.445
		5. Infeasible	0.000	0.040	0.040	0.955	0.001	0.030	0.030	0.940
	(c)	1. Proposed	0.004	0.045	0.045	0.985	0.002	0.044	0.044	0.940
		2. Naive	0.131	0.009	0.132	0.000	0.131	0.006	0.131	0.000
		3. 2SLS	0.008	0.057	0.057	0.890	0.007	0.040	0.041	0.875
		4. 2SRI	0.060	0.055	0.082	0.680	0.060	0.037	0.071	0.475
		5. Infeasible	0.001	0.051	0.051	0.945	−0.002	0.039	0.039	0.950
	(d)	1. Proposed	0.005	0.057	0.057	0.960	0.002	0.044	0.044	0.935
		2. Naive	0.137	0.009	0.137	0.000	0.136	0.006	0.137	0.000
		3. 2SLS	0.276	3.211	3.222	0.860	0.057	1.533	1.534	0.880
		4. 2SRI	0.058	0.056	0.081	0.640	0.059	0.036	0.070	0.500
		5. Infeasible	0.003	0.056	0.056	0.950	−0.001	0.038	0.038	0.950
Hard to identify	(a)	1. Proposed	0.004	0.034	0.034	0.985	0.003	0.027	0.027	0.985
		2. Naive	−0.026	0.012	0.029	0.445	−0.027	0.009	0.028	0.105
		3. 2SLS	0.016	0.051	0.054	0.950	0.016	0.034	0.037	0.935
		4. 2SRI	0.029	0.023	0.037	0.765	0.027	0.015	0.032	0.675
		5. Infeasible	−0.002	0.042	0.042	0.970	0.001	0.030	0.030	0.965
	(b)	1. Proposed	0.003	0.036	0.036	0.995	0.002	0.028	0.028	0.975
		2. Naive	−0.022	0.011	0.025	0.495	−0.022	0.008	0.024	0.220
		3. 2SLS	4.689	66.751	66.915	0.970	0.034	0.250	0.253	0.935
		4. 2SRI	0.028	0.023	0.037	0.750	0.029	0.016	0.033	0.525
		5. Infeasible	−0.001	0.041	0.041	0.950	0.000	0.030	0.030	0.970
	(c)	1. Proposed	0.006	0.046	0.046	0.990	−0.004	0.036	0.036	0.980
		2. Naive	−0.025	0.013	0.028	0.535	−0.026	0.009	0.027	0.165
		3. 2SLS	0.018	0.048	0.052	0.930	0.015	0.033	0.036	0.905
		4. 2SRI	0.061	0.047	0.077	0.720	0.058	0.032	0.066	0.540
		5. Infeasible	0.003	0.055	0.055	0.960	−0.001	0.040	0.040	0.970
	(d)	1. Proposed	0.002	0.050	0.050	0.985	−0.006	0.039	0.039	0.975
		2. Naive	−0.020	0.012	0.024	0.665	−0.021	0.009	0.023	0.345
		3. 2SLS	−0.101	1.727	1.730	0.960	0.005	0.133	0.133	0.935
		4. 2SRI	0.061	0.048	0.077	0.690	0.058	0.030	0.066	0.500
		5. Infeasible	0.001	0.055	0.055	0.945	−0.004	0.039	0.039	0.960

Abbreviations: Bias, empirical standard error (ESE), root mean squared error (RMSE), and coverage probability (CP) of the estimated log‐hazard ratio in 200 iterations by estimation methods (“Method” column) are summarized. (a): Strong common predictor and strong confounder; (b): Weak common predictor and strong confounder; (c): Strong common predictor and weak confounder; (d): Weak common predictor and weak confounder.

Our proposed method also demonstrates good CP, which is somewhat conservative but stable when compared to the infeasible method. These results suggest that our proposed method performs well in both the estimation and inference of HRs within the CPHM when there is a clustered structure among unmeasured confounders.

Some additional simulation experiments are presented in Appendix B. These results suggest that our proposed method is robust to violations of the exclusion restriction. It also demonstrates some robustness to misspecification of the distribution of the treatment variable; however, substantial bias may arise in certain situations. As is generally the case in Bayesian inference, careful consideration and diagnosis of the model are still necessary [39]. When continuous unmeasured confounders exist, our proposed method may also yield substantial bias. While the assumption of cluster‐structured data is crucial for adjusting for unmeasured confounders that arise from the cluster structure, it is important to note that unmeasured confounders are typically unobservable. Thus, assuming a clustered structure should be viewed as one of several possible modeling strategies.

## 5 UK Biobank Data Analysis

As a demonstration, we perform a real data analysis to evaluate the performance of our proposed method in practical scenarios. Specifically, we use UK Biobank data to investigate the causal effect of dietary habits on the incidence of cardiovascular disease. Additional information is provided in Appendix C.

For the exposure variable, we use “fruit intake” data collected from the 24‐h dietary recall questionnaire (Resource: 118240). To identify SNPs related to fruit intake, we reviewed previous studies and identified 20 SNPs associated with the exposure variable [40, 41, 42, 43]. For the outcome variable, we define CVD as the occurrence of “Myocardial Infarction (MI)” in Algorithmically‐defined outcomes (ADOs) version 2.

In the analysis, we perform a complete‐case analysis using subjects who did not experience any CVD events before the baseline date. The analysis population consists of 128,276 subjects. Since individual SNPs are only weakly correlated with the exposure variable, an allele score, a weighted linear combination of SNPs, is constructed for use in IV methods [2] and our proposed method. An allele score is used to construct a “strong” IV by combining multiple weak IVs. To estimate the weights for the allele score, we split the analysis dataset [2]. Specifically, the dataset is divided in a one‐to‐three ratio: three‐quarters of the dataset are used to estimate the weights, while the remaining quarter is used for the subsequent main analysis.

The analysis is conducted using six methods. The first three are ordinary CPHM approaches:

1 no adjustment for any covariates,
2 adjustment for age and sex, and
3 adjustment for age, sex, smoking habits, and drinking habits [44, 45].

Note that these methods are performed using the remaining quarter of the dataset. The last two IV methods: 2SLS, 2SRI, and the proposed method using the allele score. Note that the role of the allele score differs between the two IV methods and the proposed method (see Section 2.4). For 2SLS, only the exposure model includes age and sex as covariates, whereas for 2SRI and the proposed method, both the exposure and outcome models include age and sex. In the proposed method, cluster construction is applied only to the intercept of the exposure model, $\alpha_{0k}$.

The analysis results are summarized in Table 2. No significant causal effects of fruit intake on CVD are observed across all models. However, the point estimates exhibit differing tendencies. For the two ordinary IV methods, the estimated HR exceeds 1, whereas for the other methods, it does not. Additionally, the two IV methods have excessively wide CIs, large errors in the point estimates, consistent with the tendencies observed in the simulation experiments. Note that the influence of the additional two covariates, smoking habits and drinking habits, on HR is limited compared to the findings of the previous study [45].

**TABLE 2:** Estimated Hazard Ratio in UK Biobank data analysis.

Methods	Point estimate (95% CI)
CPHM (No adjustment)	0.976(0.943,1.011)
CPHM (Age and sex)	0.978(0.944,1.014)
CPHM (Age, sex, smoking and drinking habits)^a^	0.981(0.947,1.017)
2SLS^b^	1.254(0.994,1.583)
2SRI^c^	1.548(0.518,4.627)
Proposed^c^	0.907(0.769,1.031)

Abbreviation: CPHM, Cox proportional hazard model.

^a^ Nine subjects were excluded from the analysis due to missing data.

^b^ Age and sex are included as covariates in the exposure model.

^c^ Age and sex are included as covariates in both the exposure and outcome models.

In our proposed Bayesian method, as described in Appendix C, we consider that the analysis dataset can be divided into two clusters. The clusters are identified using posterior medians of $\alpha_{0k}$ for subjects. One cluster, representing the majority, consists of subjects who tend to eat few fruits and show no causal effects; mean (SD) of the exposure variable is 1.97(1.44), and point estimate (SE) of estimated HR by the ordinary CPHM is 0.91(0.03). The other cluster, representing the minority, consists of subjects who tend to eat many fruits and exhibit large causal effects; mean (SD) of the exposure variable is 7.11(1.79), and point estimate (SE) of estimated HR by the ordinary CPHM is 0.64(0.12). The former (majority) group can be characterized as the “Health‐unconscious” group, while the latter (minority) group can be characterized as the “Health‐conscious” group. The characteristics of these groups are summarized in Table 3. This classification appears to be reasonably accurate: compared to the Health‐unconscious group, the Health‐conscious group consists primarily of females and has lower rates of smoking and drinking habits, as well as better sleep quality. In particular, drinking habits show clear differences between the groups. Note that this data analysis is only illustrative; to clarify the causal effect we considered, more sophisticated analyses would be required.

**TABLE 3:** Summary of group characteristics in UK Biobank data analysis. For continuous variable, mean (SD). For categorical variable, the number of subjects (%).

	Group		
	Health‐unconscious	Health‐conscious
Parameter	N=30025	N=2044	SMD
Age	56.4 (8.0)	56.8 (7.9)	0.050
Sex	13341 (44.4%)	833 (40.8%)	0.073
Smoking habits	2173 (7.2%)	102 (5.0%)	0.092
Drinking habits	28218 (94.0%)	1845 (90.3%)	0.138
Sleeping status^a^	1951 (19.4%)	153 (23.4%)	0.098

*Note*: Sex: male = 1; female = 0; Smoking habits: current smoker = 1; previous or never smoked = 0; Drinking habits: Current drinker = 1; previous or never drank = 0; Sleeping status: Between 3 and 9 h = 1; Less than 3 h or more than 9 h = 0.

Abbreviation: SMD, standardized mean differences.

^a^ There are approximately 68.5% missing subjects.

## 6 Discussions and Conclusions

In this study, we proposed a novel nonparametric Bayesian method employing the general Bayes techniques to estimate hazard ratios (HRs) within the Cox Proportional Hazards Model, particularly in scenarios involving unmeasured confounders. Our method identifies clusters based on the likelihood of exposure and outcome variables and estimates HRs by integrating the likelihoods across clusters, effectively adjusting for the effects of unmeasured confounders. The results from the simulation studies and real data analysis demonstrate the potential of our approach in addressing challenges posed by unmeasured confounders, thereby providing more reliable causal effect estimates in observational studies.

A key assumption of our method is that unmeasured confounders influence outcomes through cluster effects. As discussed in Section 2, this assumption is reasonable under certain conditions and offers a pragmatic solution when direct identification of unmeasured variables is infeasible. However, this data structure is fundamental to our proposed method, and the method may not perform well when the assumption is violated. Future research should aim to relax this assumption and develop more flexible frameworks that can accommodate a wider range of data structures and confounder characteristics.

As discussed in Section 2.3, a common predictor shared across clusters can be viewed as an instrumental variable (IV), which plays a significant role in facilitating cluster detection in our method. Importantly, violations of the exclusion restriction are acceptable. Additionally, in our proposed method, the assumption of homogeneous treatment effects [12, 19], which is commonly assumed in some IV methods, may be relaxed. The model (2.2) can be interpreted as a frailty model [13], as the baseline hazard functions vary across clusters. In this model, the influence of the cluster effect on the outcome is mediated solely through the baseline hazard function $\lambda_{0i}$, rather than through the treatment effects $\beta_{a}$. This aligns with the assumption of homogeneous treatment effects [2]. Furthermore, model (2.2) may be extended to account for heterogeneous treatment effects, allowing the treatment effect $\beta_{ai}$ to vary across clusters. While this relaxation introduces greater flexibility, it may complicate the interpretation of parameters. These considerations are discussed in detail in Appendix D. Overall, our method provides a promising solution to some of the limitations associated with traditional IV approaches.

## Author Contributions

S.O. wrote the article and made the main contributions to this study. Additionally, S.O. conducted the simulation experiments and UK Biobank data analysis. S.S., T.O., and T.N. provided comments on the proposed method from a Bayesian perspective. T.O. also supported some of the simulation experiments. K.H., the principal investigator of the UK Biobank Resource under Application Number 148863, prepared the analysis dataset and provided comments on the UK Biobank data analysis from a medical perspective. M.T. contributed comments from both statistical and medical perspectives.

## Funding

This work was supported by the Japan Society for the Promotion of Science (Grant Nos. 23K13019, 23K20592, 24K14862, 24K20739, and 25K21166).

## Conflicts of Interest

The authors declare no conflicts of interest.

## Supporting information




**Data S1.** sim70360‐sup‐0001‐Supinfo.pdf.

## Data Availability

This research has been conducted using the UK Biobank Resource under Application Number 148863. The data supporting this study's findings are available from the UK Biobank but are not publicly accessible due to licensing restrictions. However, they can be obtained from the authors upon reasonable request with permission from the UK Biobank.

## References

[1] M. A. Brookhart and S. Schneeweiss , “Preference‐Based Instrumental Variable Methods for the Estimation of Treatment Effects: Assessing Validity and Interpreting Results,” International Journal of Biostatistics 3 (2007): 14. 19655038 10.2202/1557-4679.1072 PMC2719903

[2] S. Burgess and S. G. Thompson , Mendelian Randomization: Methods for Causal Inference Using Genetic Variants (CRC Press, 2021).

[3] D. A. Jenkins , K. H. Wade , D. Carslake , et al., “Estimating the Causal Effect of BMI on Mortality Risk in People With Heart Disease, Diabetes and Cancer Using Mendelian Randomization,” International Journal of Cardiology 330 (2021): 214–220. 33592239 10.1016/j.ijcard.2021.02.027

[4] K. H. Wade , D. Carslake , N. Sattar , G. Davey Smith , and N. J. Timpson , “BMI and Mortality in UK Biobank: Revised Estimates Using Mendelian Randomization,” Obesity 26 (2018): 1796–1806. 30358150 10.1002/oby.22313 PMC6334168

[5] D. R. Cox , “Regression Models and Life‐Tables,” Journal of the Royal Statistical Society. Series B, Statistical Methodology 34 (1972): 187–202.

[6] B. Kianian , J. In Kim , J. P. Fine , and L. Peng , “Causal Proportional Hazards Estimation With a Binary Instrumental Variable,” Statistica Sinica 31 (2021): 673–699. 34970068 10.5705/ss.202019.0096 PMC8716008

[7] L. Wang , E. Tchetgen Tchetgen , T. Martinussen , and S. Vansteelandt , “Instrumental Variable Estimation of the Causal Hazard Ratio,” Biometrics 79 (2023): 539–550. 36377509 10.1111/biom.13792

[8] Y. Cui , H. Michael , F. Tanser , and E. Tchetgen Tchetgen , “Instrumental Variable Estimation of the Marginal Structural Cox Model for Time‐Varying Treatments,” Biometrika 110 (2023): 101–118. 36798841 10.1093/biomet/asab062 PMC9919489

[9] M. A. Hernán and J. M. Robins , Causal Inference: What if (Chapman & Hill/CRC, 2020).

[10] D. R. Ragland , “Dichotomizing Continuous Outcome Variables: Dependence of the Magnitude of Association and Statistical Power on the Cutpoint,” Epidemiology 3 (1992): 434–440. 1391136 10.1097/00001648-199209000-00009

[11] J. V. Terza , A. Basu , and P. J. Rathouz , “Two‐Stage Residual Inclusion Estimation: Addressing Endogeneity in Health Econometric Modeling,” Journal of Health Economics 27 (2008): 531–543. 18192044 10.1016/j.jhealeco.2007.09.009 PMC2494557

[12] P. Martínez‐Camblor , T. Mackenzie , D. O. Staiger , P. P. Goodney , and A. J. O Malley , “Adjusting for Bias Introduced by Instrumental Variable Estimation in the Cox Proportional Hazards Model,” Biostatistics 20 (2019): 80–96. 29267847 10.1093/biostatistics/kxx062

[13] G. G. Nielsen , R. D. Gill , P. K. Andersen , and T. I. A. Sørensen , “A Counting Process Approach to Maximum Likelihood Estimation in Frailty Models,” Scandinavian Journal of Statistics 19 (1992): 25–43.

[14] C. N. Basu Anirban and G. Chapman Cole , Comparing 2SLS VS 2SRI for Binary Outcomes and Binary Exposures Tech. Rep (National Bureau of Economic Research, 2017).

[15] F. Wan , D. Small , and N. Mitra , “A General Approach to Evaluating the Bias of 2‐Stage Instrumental Variable Estimators,” Statistics in Medicine 37 (2018): 1997–2015. 29572890 10.1002/sim.7636

[16] S. Orihara , A. Goto , and M. Taguri , “Instrumental Variable Estimation of Causal Effects With Applying Some Model Selection Procedures Under Binary Outcomes,” Behaviormetrika 50 (2023): 241–262.

[17] S. Orihara and A. Goto , “Comparison of Instrumental Variable Methods With Continuous Exposure and Binary Outcome: A Simulation Study,” Journal of Epidemiology 35, no. 1 (2025): 11–20. 38644194 10.2188/jea.JE20230271 PMC11637812

[18] J. M. Wooldridge , “Quasi‐Maximum Likelihood Estimation and Testing for Nonlinear Models With Endogenous Explanatory Variables,” Journal of Econometrics 182 (2014): 226–234.

[19] S. Burgess , D. S. Small , and S. G. Thompson , “A Review of Instrumental Variable Estimators for Mendelian Randomization,” Statistical Methods in Medical Research 26 (2017): 2333–2355. 26282889 10.1177/0962280215597579 PMC5642006

[20] G. Sandeep , D. G. M. Fabiola , M. Stein Catherine , and Z. Andreas , “Mendelian Randomization,” in Statistical Human Genetics: Methods and Protocols, edited by R. C. Elston (Springer, 2017), 581–628.

[21] S. Orihara , S. Fukuma , T. Ikenoue , and M. Taguri , “Likelihood‐Based Instrumental Variable Methods for Cox Proportional Hazards Model,” Japanese Journal of Statistics and Data Science 8, no. 1 (2024): 393–424.

[22] M. D. Escobar and M. West , “Computing Nonparametric Hierarchical Models,” in Practical Nonparametric and Semiparametric Bayesian Statistics (Springer, 1998), 1–22.

[23] T. G. Conley , C. B. Hansen , R. E. McCulloch , and P. E. Rossi , “A Semi‐Parametric Bayesian Approach to the Instrumental Variable Problem,” Journal of Econometrics 144 (2008): 276–305.

[24] P. Müller , F. A. Quintana , A. Jara , and T. Hanson , Bayesian Nonparametric Data Analysis, vol. 1 (Springer, 2015).

[25] P. G. Bissiri , C. C. Holmes , and S. G. Walker , “A General Framework for Updating Belief Distributions,” Journal of the Royal Statistical Society. Series B, Statistical Methodology 78 (2016): 1103–1130. 27840585 10.1111/rssb.12158 PMC5082587

[26] Y. Xu , J. S. Kim , L. K. Hummers , A. A. Shah , and S. L. Zeger , “Causal Inference Using Multivariate Generalized Linear Mixed‐Effects Models,” Biometrics 80 (2024): ujae100. 39319549 10.1093/biomtc/ujae100 PMC11422711

[27] C. Pan and B. Cai , “A Bayesian Semiparametric Mixed Effects Proportional Hazards Model for Clustered Partly Interval‐Censored Data,” Statistical Modelling 24 (2024): 459–479.

[28] W. Miao , Z. Geng , and E. J. Tchetgen Tchetgen , “Identifying Causal Effects With Proxy Variables of an Unmeasured Confounder,” Biometrika 105 (2018): 987–993. 33343006 10.1093/biomet/asy038 PMC7746017

[29] X. Shi , W. Miao , J. C. Nelson , and E. J. Tchetgen Tchetgen , “Multiply Robust Causal Inference With Double‐Negative Control Adjustment for Categorical Unmeasured Confounding,” Journal of the Royal Statistical Society. Series B, Statistical Methodology 82 (2020): 521–540. 33376449 10.1111/rssb.12361 PMC7768794

[30] H. M. Kang , N. A. Zaitlen , C. M. Wade , et al., “Efficient Control of Population Structure in Model Organism Association Mapping,” Genetics 178 (2008): 1709–1723. 18385116 10.1534/genetics.107.080101 PMC2278096

[31] J. B. Copas and H. G. Li , “Inference for Non‐Random Samples,” Journal of the Royal Statistical Society. Series B, Statistical Methodology 59 (1997): 55–95.

[32] N. M. Davies , M. V. Holmes , and G. D. Smith , “Reading Mendelian Randomisation Studies: A Guide, Glossary, and Checklist for Clinicians,” BMJ 362 (2018): k601. 30002074 10.1136/bmj.k601 PMC6041728

[33] D. Sinha , J. G. Ibrahim , and M.‐H. Chen , “A Bayesian Justification of Cox's Partial Likelihood,” Biometrika 90 (2003): 629–641.

[34] J. D. Kalbfleisch and R. L. Prentice , The Statistical Analysis of Failure Time Data (John Wiley & Sons, 2011).

[35] N. Reid , “On Partial Likelihood,” Journal of the Royal Statistical Society. Series A, Statistics in Society 187 (2024): 567–577.

[36] D. A. Stephens , W. S. Nobre , E. E. M. Moodie , and A. M. Schmidt , “Causal Inference Under Mis‐Specification: Adjustment Based on the Propensity Score (With Discussion),” Bayesian Analysis 18 (2023): 639–694.

[37] L. Held , I. Gravestock , and D. Sabanés Bové , “Objective Bayesian Model Selection for Cox Regression,” Statistics in Medicine 35 (2016): 5376–5390. 27580645 10.1002/sim.7089

[38] P. R. Hahn , C. M. Carvalho , D. Puelz , and J. He , “Regularization and Confounding in Linear Regression for Treatment Effect Estimation,” Bayesian Analysis 13 (2018): 163–182.

[39] C. P. Robert , The Bayesian Choice: From Decision‐Theoretic Foundations to Computational Implementation (Springer, 2007).

[40] L. Chen , Y. Su , H. Li , Z. Yang , J. J. Li , and D. Xing , “The Role of Dietary Preferences in Osteoarthritis: A Mendelian Randomization Study Using Genome‐Wide Association Analysis Data From the UK Biobank,” Frontiers in Nutrition 11 (2024): 1373850. 38742020 10.3389/fnut.2024.1373850 PMC11089188

[41] X. Wang , L. Chen , R. Cao , et al., “Associations of 10 Dietary Habits With Breast Cancer: A Mendelian Randomization Study,” Frontiers in Nutrition 10 (2023): 1215220. 38075235 10.3389/fnut.2023.1215220 PMC10702979

[42] M. He , L. Huan , X. Wang , Y. Fan , and J. Huang , “Nine Dietary Habits and Risk of Colorectal Cancer: A Mendelian Randomization Study,” BMC Medical Genomics 17 (2024): 21. 38233852 10.1186/s12920-023-01782-7 PMC10795375

[43] M. Yang , X. Gao , L. Xie , et al., “Causal Associations Between Dietary Habits and CVD: A Mendelian Randomisation Study,” British Journal of Nutrition 130 (2023): 2104–2113. 37381916 10.1017/S000711452300140X

[44] P. Van't Veer , M. C. J. F. Jansen , M. Klerk , and F. J. Kok , “Fruits and Vegetables in the Prevention of Cancer and Cardiovascular Disease,” Public Health Nutrition 3 (2000): 103–107. 10786730 10.1017/s1368980000000136

[45] V. Miller , A. Mente , M. Dehghan , et al., “Fruit, Vegetable, and Legume Intake, and Cardiovascular Disease and Deaths in 18 Countries (PURE): A Prospective Cohort Study,” Lancet 390 (2017): 2037–2049. 28864331 10.1016/S0140-6736(17)32253-5

