# Supplementary material for: Nonparametric Bayesian Adjustment of Unmeasured Confounders in Cox Proportional Hazards Models
Source: Stat Med. 2026 Jan 22;45(1-2):e70360. doi: 10.1002/sim.70360 (PMC12826352; doi:10.1002/sim.70360)
Supplement: Supplementary file 1 — Data S1. sim70360‐sup‐0001‐Supinfo.pdf. [file SIM-45-0-s001.pdf]

Supplementary Material for "Nonparametric Bayesian Adjustment of Unmeasured Confounders in Cox Proportional Hazards Models"

Shunichiro Orihara[1], Shonosuke Sugasawa[2], Tomohiro Ohigashi[3], Keita Hirano[4], Tomoyuki Nakagawa[5], and Masataka Taguri[1]

[1]Department of Health Data Science, Tokyo Medical University, Tokyo, Japan
[2]Graduate School of Economics, Keio University, Tokyo, Japan
[3]Department of Information and Computer Technology, Faculty of Engineering, Tokyo University of Science, Tokyo, Japan
[4]Department of Human Health Sciences, Graduate School of Medicine, Kyoto University, Kyoto, Japan
[5]School of Data Science, Meisei University, Tokyo, Japan

Mailing address: 6-1-1 Shinjuku, Shinjuku-ku, Tokyo 160-8402, Japan

Email address: orihara@tokyo-med.ac.jp

Contents

A Additional Sampling Information **3**

A.1 Applying shrinkage techniques for $\boldsymbol{\beta}_z$ 3

A.2 Detailed sampling algorithms for S and γ 3

B Additional Information for Simulation Experiments **5**

B.1 Setting of the exposure and outcome distribution in the main manuscript . . 5

B.2 Additional information for the competitors 9

B.3 Additional table and figures in the main manuscript 9

B.4 1 shot results for proposed method 13

B.5 Additional simulation experiments 16

B.5.1 Additional scenario 1: True likelihood for exposure variable A is log-normal; analysis model is normal 16

B.5.2 Additional scenario 2: True likelihood for exposure variable A is normal; analysis model is log-normal 21

B.5.3 Additional scenario 3: Violation of the exclusion restriction assumption 24

B.5.4 Additional scenario 4: Continuous unmeasured confounders 28

B.5.5 Additional scenario 5: No cluster effects 33

C Additional Information for UK Biobank Data Analysis **36**

C.1 Likelihoods and prior distributions 36

C.2 Additional analysis results 37

D Overcoming Homogeneous Treatment Effect Assumption and its Limitations **40**

A Additional Sampling Information

A.1 Applying shrinkage techniques for $\boldsymbol{\beta}_z$

As mentioned in the main text, valid IVs are not necessarily needed to estimate the hazard ratio properly for our proposed procedure. In other words, all covariates, including $\boldsymbol{Z}$, can be included in both models (2.1) and (2.2) in the main manuscript. However, in the Mendelian Randomization context, over 100 IVs are sometimes considered. To accurately estimate the hazard ratio, applying shrinkage methods and reducing the number of parameters are beneficial.

The simulation and real data analysis in the main manuscript, we apply the horseshoe prior (Carvalho et al., 2010) to more precisely shrink the coefficient estimates near zero to zero when sampling from the posterior for $\boldsymbol{\beta}_z$. Specifically, we consider the following prior for $\pi(\boldsymbol{\beta}_z)$:

Prior for $\boldsymbol{\beta}_z$: For each $\beta_{z\ell}$,

$$\beta_{z\ell} \mid \psi_\ell \sim N(0, \psi_\ell^2 \tau_{\beta_{z\ell}}^2), \quad \psi_\ell \sim C^+(0,1),$$

where $C^+(0,1)$ is Half-Cauchy distribution.

A.2 Detailed sampling algorithms for S and γ

Using the discussion, for example in Dahl et al. (2017), the full conditional distribution of s_i is

$$P(s_i = k \mid \cdot) \propto p(S^{i\to k};\gamma)\ell_{ik}(\beta_a, \boldsymbol{\beta}_x)\phi\Big(A_i; \alpha_{0k} + \boldsymbol{z}_i^\top \boldsymbol{\alpha}_z + \boldsymbol{v}_i^\top \boldsymbol{\alpha}_{vk}, \sigma_k^2\Big), \quad k = 1, \ldots, K_n + 1,$$

where $S^{i \to k}$ denotes a sequence $s_1, \ldots, s_n$ with $s_i = k$ and the other variables set to the current assignment, and $p(S^{i \to k}; \gamma)$ is the joint probability obtained by the Chinese restaurant representation (2.5) in the main manuscript. For $k = K_n + 1$, the parameter $(\alpha_{0k}, \boldsymbol{\alpha}_{vk}, \sigma_k^2)$ are generated from their prior distribution at each MCMC iteration. In short, clustering is achieved either by creating a new cluster for subject i, or by assigning the subject to an existing cluster with some probabilities. By iterating the sampling from the posterior, clusters S are automatically created, adjusted by the hyper-parameter γ. In the simulation experiments, we assume G_0 follows a normal distribution to simplify the calculation.

In the simulation experiments, the precision parameter γ is sampled using the same strategy as Escobar and West (1998). Specifically, it is assumed that γ has a Gamma prior distribution: $\gamma \sim Gamma(a, b)$. Additionally, we introduce a latent variable (parameter) η. Under this setting, the posteriors of these variables are described as follows:

Posterior for γ

$$\gamma \mid \eta, I^* \sim \pi Gamma(a + I^*, b - \log(\eta)) + (1 - \pi) Gamma(a + I^* - 1, b - \log(\eta)),$$

where $\pi = e^\lambda/(1 + e^\lambda)$, $\lambda = (a + I^* - 1)/I^*\{b - \log(\eta)\}$, and I^* is the current cluster size.

Posterior for η

$$\eta \mid \gamma, I^* \sim \text{Beta}(a + 1, n).$$

In the simulation experiments, a and b are set as noted in the footnote of each table.

B Additional Information for Simulation Experiments

B.1 Setting of the exposure and outcome distribution in the main manuscript

The parameter settings for the exposure variable are summarized in Table B.1.

A basic outcome model is

$$\lambda(t_i \mid a_i, v_i, k) = \lambda_{0k} \exp\left\{-0.1a_i + 0.1v_i\right\}.$$

First, we explain "Easy to identify" setting. For $K = 0$ subjects, $\lambda_{0k} = 0.15$. Additionally, we consider "latent" event time, denoted as $\tilde{T}_i$ is derived from

$$\tilde{\lambda}(t_i \mid a_i, v_i, k \neq 0) = 0.005 \exp\left\{-0.1a_i + 0.1v_i\right\}. \tag{B.1}$$

If $K = 1$ and $\tilde{T}_i < 65$, $T_i = \tilde{T}_i$, the outcome model is (B.1). Else, $\lambda_{0k} = 0.15$. If $K = 2$ and $\tilde{T}_i < 40$, $T_i = \tilde{T}_i$, the outcome model is (B.1). Else, $\lambda_{0k} = 0.1$.

Next, we explain "Hard to identify" setting. For $K = 0$ subjects, $\lambda_{0k} = 0.035$. Additionally, we consider "latent" event time, denoted as $\tilde{T}_i$ is derived from

$$\tilde{\lambda}(t_i \mid a_i, v_i, k \neq 0) = 0.015 \exp\left\{-0.1a_i + 0.1v_i\right\}. \tag{B.2}$$

If $K = 1$ and $\tilde{T}_i < 65$, $T_i = \tilde{T}_i$, the outcome model is (B.2). Else, $\lambda_{0k} = 0.1$. If $K = 2$ and $\tilde{T}_i < 40$, $T_i = \tilde{T}_i$, the outcome model is (B.1). Else, $\lambda_{0k} = 0.1$.

In summary, the distribution for the exposure and outcome variables are summarized in Figure B.1–B.4.

Table B.1: Summary of simulation settings

**Setting**	**Common predictor strength**	**Confounder strength**	**Scenario #**	**Parameter settings**
Easy to identify	Strong	Strong	(a)	$\alpha_{0k} = 16 - 8I\{K=1\} - 14I\{K=2\}$<br>$\alpha_z = 1.5$<br>$\alpha_{vk} = 4 - 2I\{K=1\} - 3I\{K=2\}$
	Weak	Strong	(b)	$\alpha_{0k} = 16 - 8I\{K=1\} - 14I\{K=2\}$<br>$\alpha_z = 0.5$<br>$\alpha_{vk} = 4 - 2I\{K=1\} - 3I\{K=2\}$
	Strong	Weak	(c)	$\alpha_{0k} = 16 - 8I\{K=1\} - 14I\{K=2\}$<br>$\alpha_z = 1.5$<br>$\alpha_{vk} = 0.5(4 - 2I\{K=1\} - 3I\{K=2\})$
	Weak	Weak	(d)	$\alpha_{0k} = 16 - 8I\{K=1\} - 14I\{K=2\}$<br>$\alpha_z = 0.5$<br>$\alpha_{vk} = 0.5(4 - 2I\{K=1\} - 3I\{K=2\})$
Hard to identify	Strong	Strong	(a)	$\alpha_{0k} = 12 - 4I\{K=1\} - 9I\{K=2\}$<br>$\alpha_z = 1.5$<br>$\alpha_{vk} = 4.5 - 2.5I\{K=1\} - 3I\{K=2\}$
	Weak	Strong	(b)	$\alpha_{0k} = 12 - 4I\{K=1\} - 9I\{K=2\}$<br>$\alpha_z = 0.5$<br>$\alpha_{vk} = 4.5 - 2.5I\{K=1\} - 3I\{K=2\}$
	Strong	Weak	(c)	$\alpha_{0k} = 12 - 4I\{K=1\} - 9I\{K=2\}$<br>$\alpha_z = 1.5$<br>$\alpha_{vk} = 0.5(4.5 - 2.5I\{K=1\} - 3I\{K=2\})$
	Weak	Weak	(d)	$\alpha_{0k} = 12 - 4I\{K=1\} - 9I\{K=2\}$<br>$\alpha_z = 0.5$<br>$\alpha_{vk} = 0.5(4.5 - 2.5I\{K=1\} - 3I\{K=2\})$

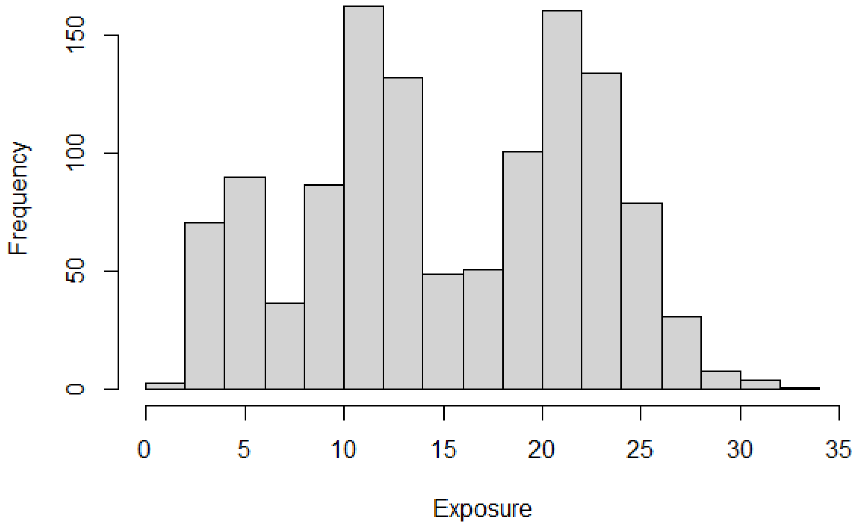

Figure B.1: Histogram of the exposure variable (crude analysis) in a simulation dataset under "Easy to identify" setting

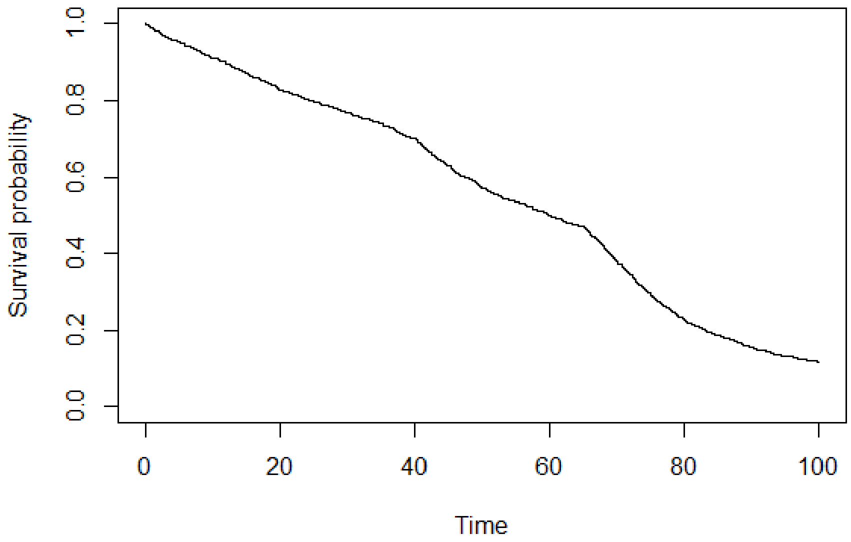

Figure B.2: Kaplan-Meier plot of the outcome variable (crude analysis) in a simulation dataset under "Easy to identify" setting

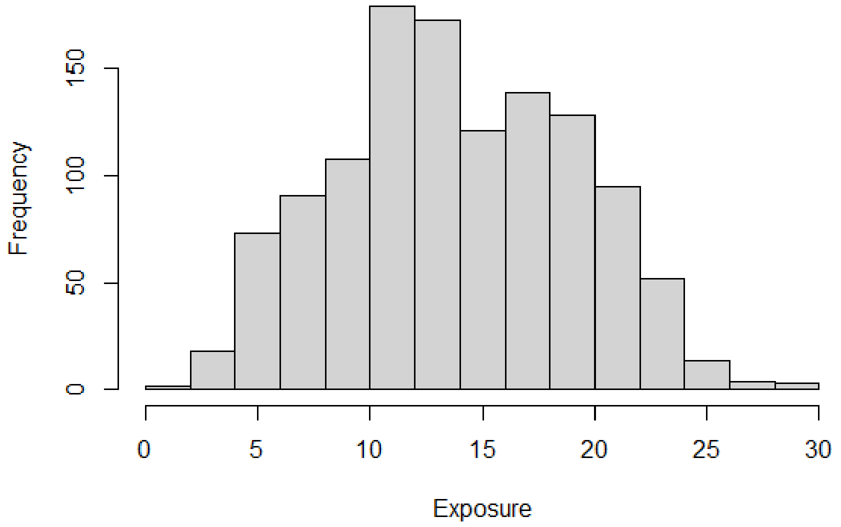

Figure B.3: Histogram of the exposure variable (crude analysis) in a simulation dataset under “Hard to identify” setting

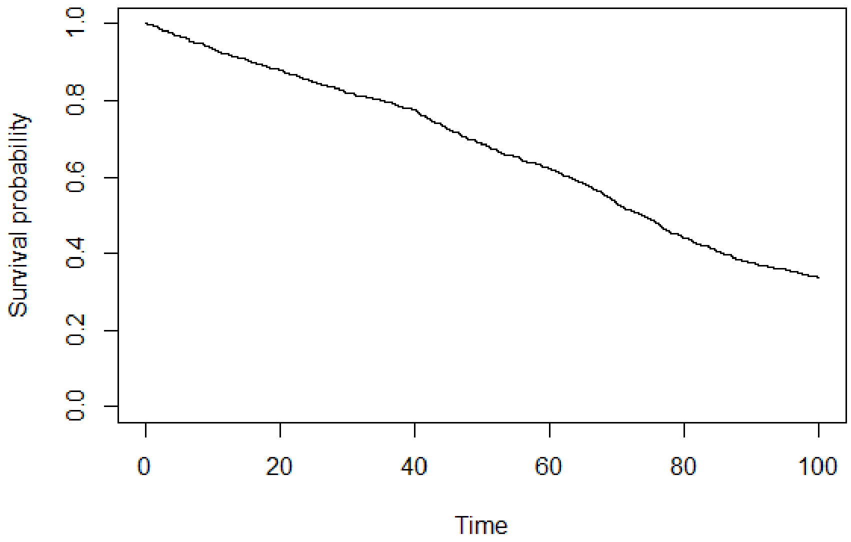

Figure B.4: Kaplan-Meier plot of the outcome variable (crude analysis) in a simulation dataset under “Hard to identify” setting

B.2 Additional information for the competitors

The proposed procedure was implemented as described in the preceding sections. In this simulation experiment, the variance parameter is homogeneous (i.e., $\sigma_k \equiv \sigma$). Therefore, in the "Sampling of Σ" step described in Section 3.2, σ is sampled from the common posterior for the variance parameter. The iteration time of the MCMC sample is 1200; the first 200 are the burn-in period, and the rest of 1000 is the posterior sample.

Four reference methods were used in this study. The first method, called the naive estimator or simply "Naive," is based on the ordinary CPHM but ignores the cluster structure (i.e., K). This approach leads to biased hazard ratio estimates. The second method, called the infeasible estimator or simply "Infeasible," includes the cluster structure (i.e., K) in the ordinary CPHM. Although this estimator is consistent and efficient, it cannot be implemented practically; thus, it is referred to as an infeasible estimator. These results are presented only as references. Both the naive and infeasible estimators were implemented using the "coxph" function of the survival library in R.

The third method, called "2SLS," is the ordinary two-stage least squares procedure for estimating the hazard ratio. Specifically, the first stage model is treated as an ordinary linear model. In the second step, predictors of the exposure variables are applied to the "coxph" function. The standard error of β_a is directly used to estimate the confidence interval.

The fourth method, called "2SRI," represents a significant alternative proposed by Martínez-Camblor et al. (2019). In simulation experiments, the first stage model is the same as 2SLS. For the second stage model, the "coxph" function, with the normal frailty option, is employed. The standard error of β_a is also directly used to estimate the confidence interval.

B.3 Additional table and figures in the main manuscript

Table B.2: Summary of Estimates for the Proposed Procedure: The iteration time is 200. The true values of "Other regression coefficients for the outcome model" (β_z and β_v) and "Variance parameter" are $(0, 0.5)$ and 0.5, respectively. In Scenarios (a) and (c), the true value of "The regression coefficient for the exposure model" (α_z) is 1.5, whereas it is 0.5 in Scenarios (b) and (d). Bias, empirical standard error (ESE), root mean squared error (RMSE), and coverage probability (CP) of the estimated log-hazard ratio in 200 iterations by estimation methods ("Method" column) are summarized.

**Setting**	**Scenario**	**Other regression coefficients for the outcome model (β_z and β_v)**								**The regression coefficient for the exposure model (α_z)**				**Variance parameter**			
		$n = 600$				$n = 1200$				$n = 600$		$n = 1200$		$n = 600$		$n = 1200$	
		**Mean**	**ESE**	**Mean**	**ESE**	**Mean**	**ESE**	**Mean**	**ESE**	**Mean**	**ESE**	**Mean**	**ESE**	**Mean**	**ESE**	**Mean**	**ESE**
Easy to identify	(a)	0.085	0.108	0.498	0.095	0.083	0.089	0.465	0.100	1.567	0.050	1.584	0.308	0.554	0.165	0.698	2.972
	(b)	0.082	0.106	0.473	0.102	0.088	0.089	0.450	0.155	0.599	0.392	0.598	0.485	0.753	2.776	0.817	4.610
	(c)	0.092	0.078	0.539	0.137	0.092	0.073	0.512	0.126	1.568	0.047	1.677	0.792	0.606	0.224	1.490	6.763
	(d)	0.090	0.096	0.502	0.113	0.094	0.075	0.446	0.080	0.655	0.572	0.681	0.734	1.283	4.833	1.454	6.104
Hard to identify	(a)	0.093	0.109	0.519	0.100	0.094	0.092	0.477	0.115	1.558	0.046	1.543	0.030	0.511	0.172	0.486	0.180
	(b)	0.093	0.114	0.489	0.137	0.100	0.087	0.435	0.097	0.555	0.053	0.543	0.033	0.535	0.288	0.511	0.354
	(c)	0.089	0.086	0.547	0.103	0.103	0.073	0.509	0.225	1.556	0.048	1.547	0.041	0.534	0.065	0.555	0.398
	(d)	0.095	0.092	0.501	0.104	0.106	0.073	0.468	0.128	0.557	0.049	0.546	0.030	0.569	0.318	0.496	0.065

(a): Strong common predictor and strong confounder; (b): Weak common predictor and strong confounder;

(c): Strong common predictor and weak confounder; (d): Weak common predictor and weak confounder.

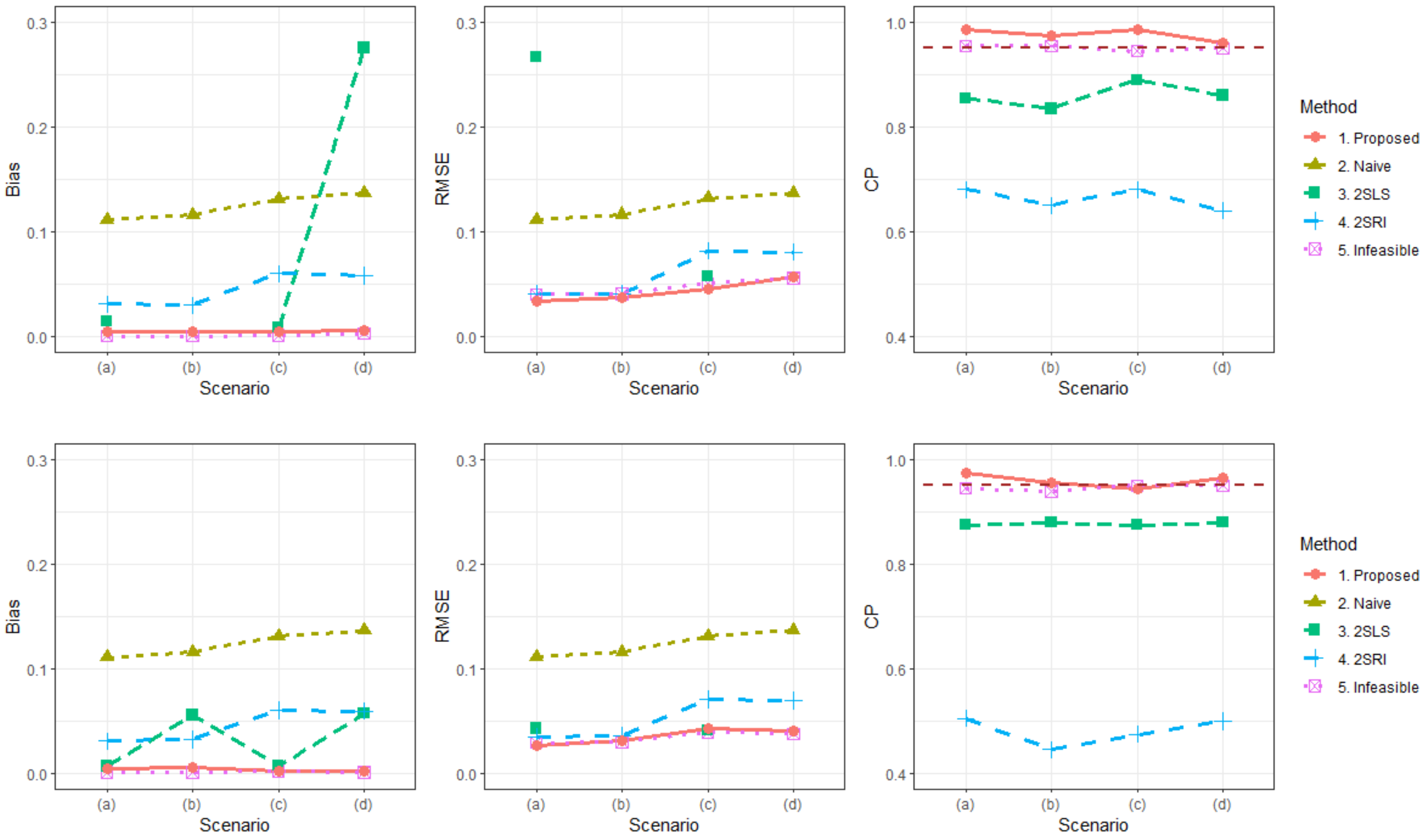

Figure B.5: Some plots of statistics for each method in “Easy to identify” setting: The iteration time is 200. Bias, root mean squared error (RMSE), and coverage probability (CP) of the estimated hazard ratio in 200 iterations by estimation methods are summarized.

Upper figure: the sample is 600; lower figure: the sample is 1200;

Extreme values are not plotted: some Bias and RMSE of 2SLS, and CP of Naive estimator;

(a): Strong common predictor and strong confounder; (b): Weak common predictor and strong confounder;

(c): Strong common predictor and weak confounder; (d): Weak common predictor and weak confounder.

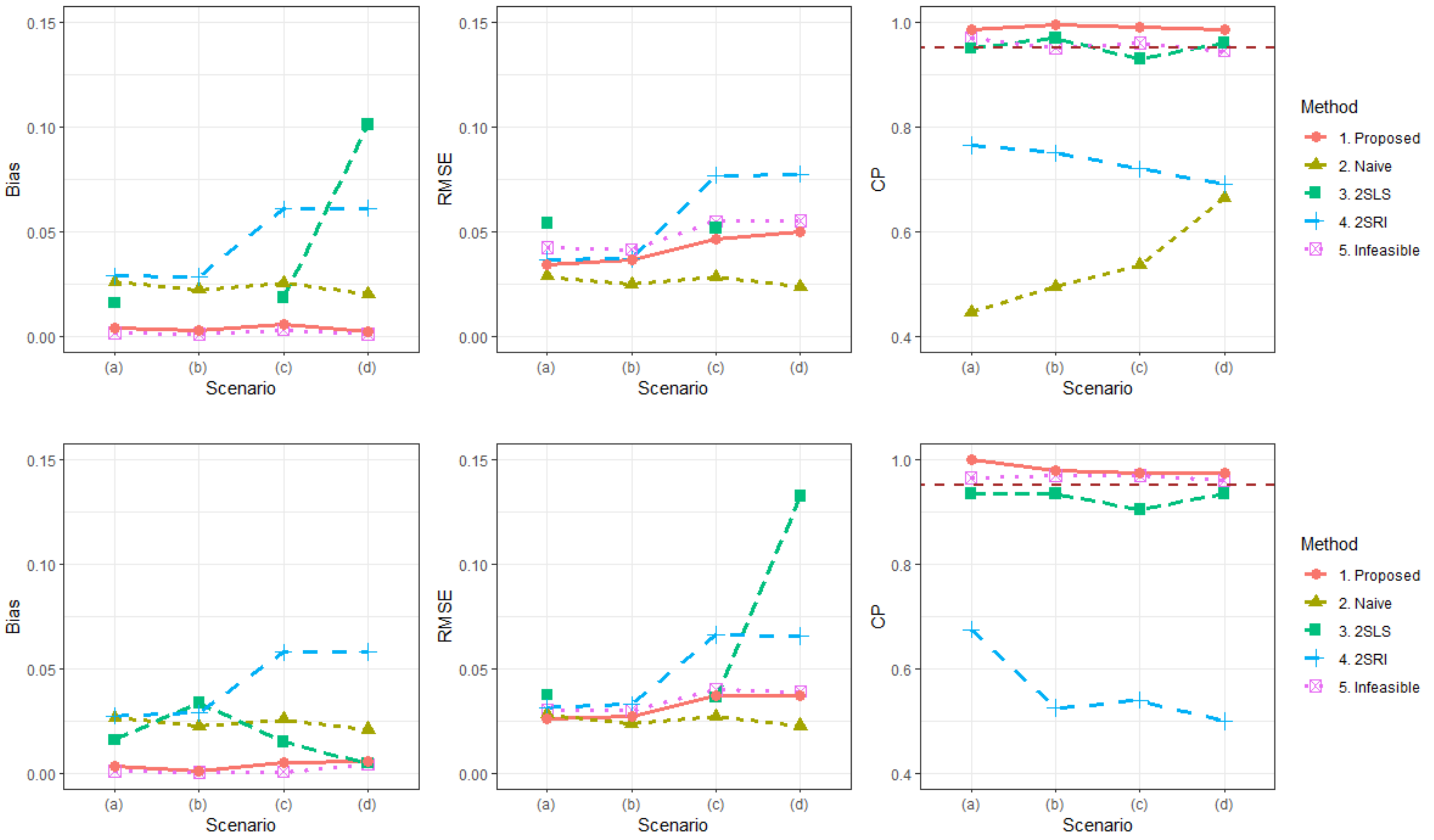

Figure B.6: Some plots of statistics for each method in "Hard to identify" setting: The iteration time is 200. Bias, root mean squared error (RMSE), and coverage probability (CP) of the estimated hazard ratio in 200 iterations by estimation methods are summarized.

Upper figure: the sample is 600; lower figure: the sample is 1200;

Extreme values are not plotted: some Bias and RMSE of 2SLS, and CP of Naive estimator;

(a): Strong common predictor and strong confounder; (b): Weak common predictor and strong confounder;

(c): Strong common predictor and weak confounder; (d): Weak common predictor and weak confounder.

B.4 1 shot results for proposed method

Additionally summary results, we also consider 1–shot results; especially focused on "Hard to identify" setting and scenario (a). Results are summarized in Figure B.7. Effective sample size of β_a, β_z, β_v, α_z, and σ^2 is 443.54, 388.90, 397.54, 116.17, and 383.77, respectively. As expected, sampling from the posterior works well, especially for the coefficients in the outcome model.

Regarding the construction of clusters, nearly exact clustering is achieved, though the number of clusters is quite large (Figure B.8). This phenomenon is well-known in DP contexts (Miller and Harrison, 2013). In this manuscript, identifying the exact clusters is not the main objective; therefore, this phenomenon is acceptable.

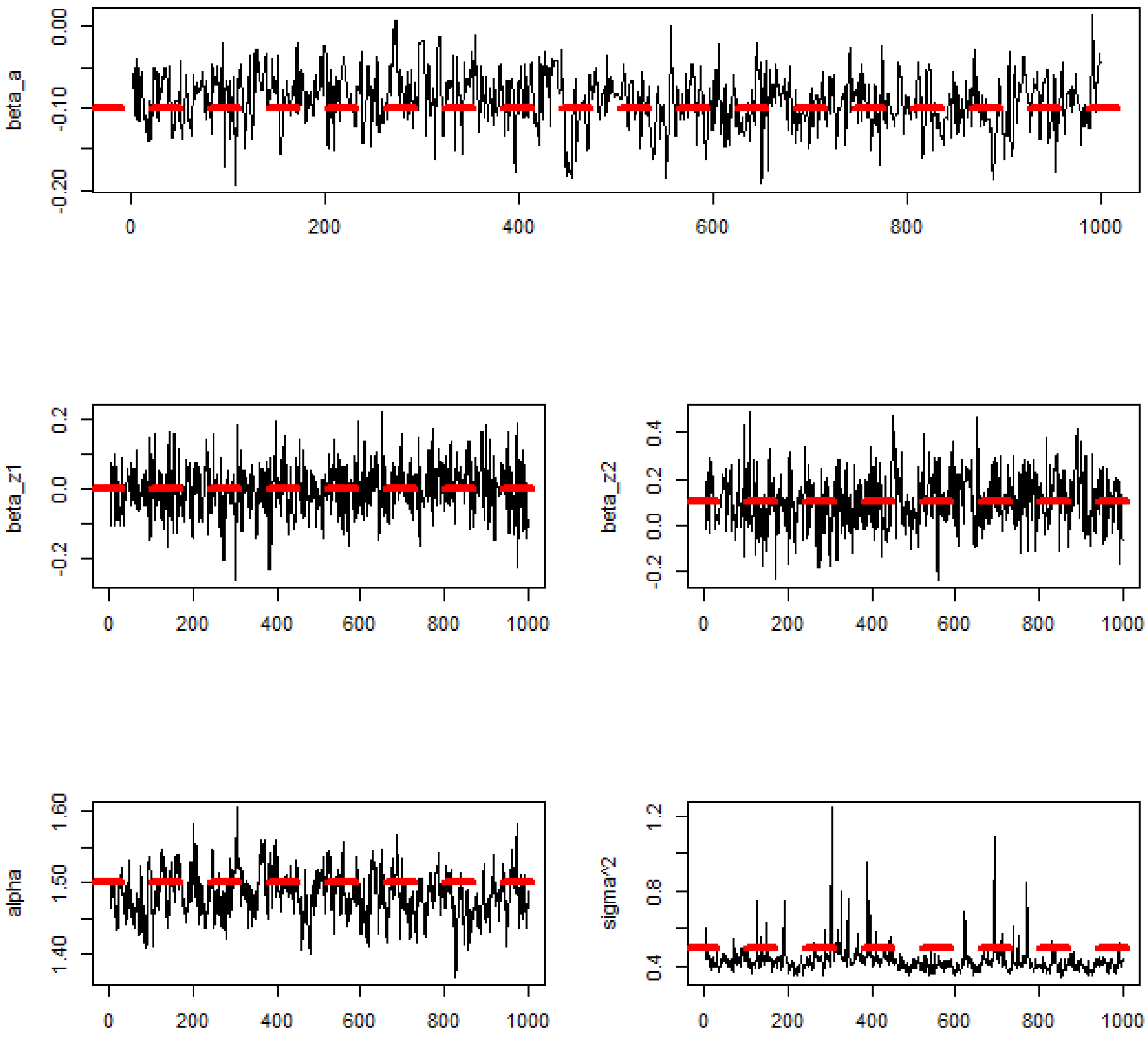

Figure B.7: Sampling plots for proposed procedure under “Hard to identify” setting and Scenario (a) in a dataset. The true values are red dashed lines.

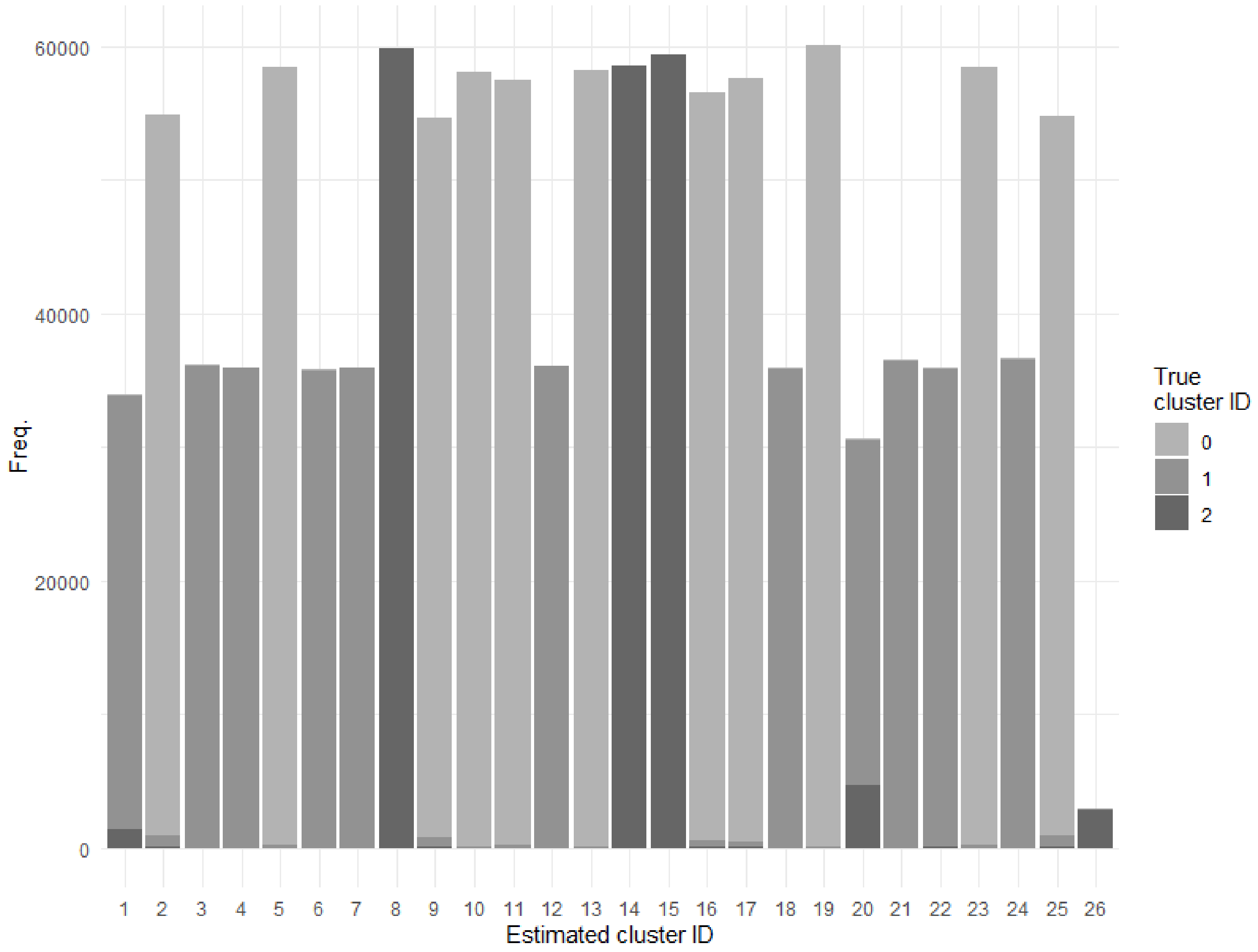

Figure B.8: Stacked bar chart of constructed clusters in “Hard to identify” setting and Scenario (a).

B.5 Additional simulation experiments

In this section, we consider several alternative simulation settings to assess the robustness of our proposed method. Specifically, we examine the following additional four scenarios, which are modifications of the "easy-to-identify" setting in the main manuscript.

B.5.1 Additional scenario 1: True likelihood for exposure variable A is log-normal; analysis model is normal

We consider a non-normal distribution for the treatment variable. Specifically, we assume a log-normal distribution: the linear predictor is the same as in Table B.1, and the error term is defined as $\varepsilon_i = \exp\{\varepsilon_i'\} - \exp\{\sigma^2/2\}$ (see Figure B.9), where

- Slightly misspecified: $\sigma = \sqrt{0.5}$
- Greatly misspecified: $\sigma = 1$.

In the former setting, the variance is 1.03 and the skewness is 2.95. In the latter setting, the variance is 2.16 and the skewness is 6.05. We use this scenario to assess how misspecifying the likelihood for the treatment variable affects the parameter of interest, β_a.

The results are summarized in Table B.3, and Figures B.10 and B.11. In the "Slightly Misspecified" setting, the proposed estimator performs almost identically to that in the main manuscript. This result suggests that our proposed method exhibits robustness to misspecification of the treatment model. In the "Greatly Misspecified" setting, the bias in the estimated treatment effect β_a increases; however, it remains obviously small compared to the results of "Additional Scenario 2."

A summary of this scenario is presented along with that of "Additional Scenario 2."

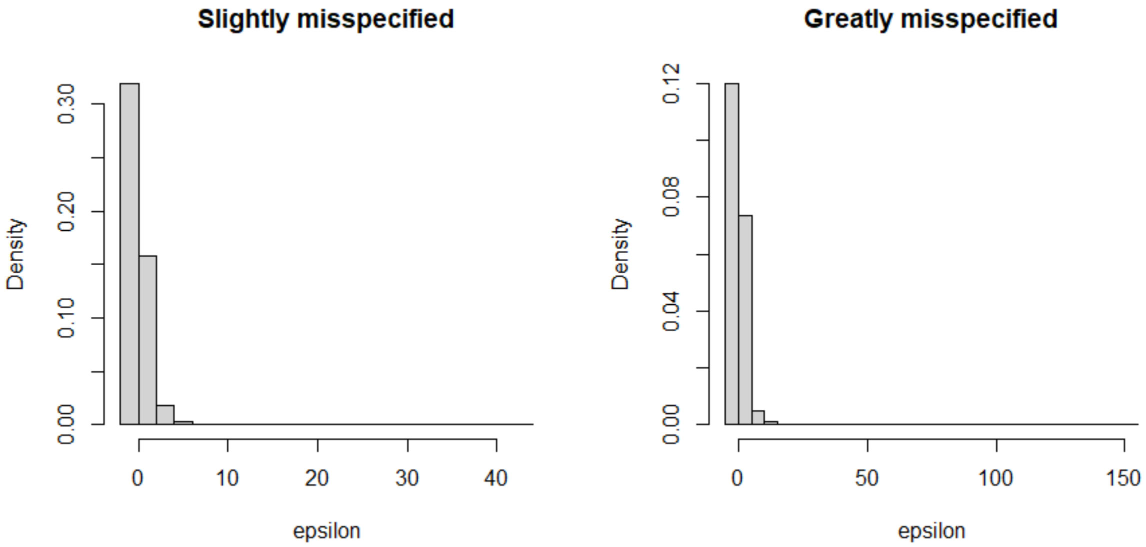

Figure B.9: Histogram of error distributions

Table B.3: Summary of Hazard Ratio Estimates under additional scenario 1: The iteration time is 200, and the true values of the log hazard ratio is −0.1. Bias, empirical standard error (ESE), root mean squared error (RMSE), and coverage probability (CP) of the estimated log-hazard ratio in 200 iterations by estimation methods ("Method" column) are summarized.

			**log–hazard ratio**							
			$n = 600$				$n = 1200$			
**Setting**	**Scenario**	**Method**	**Bias**	**ESE**	**RMSE**	**CP**	**Bias**	**ESE**	**RMSE**	**CP**
Slightly misspecified	(a)	1. Proposed	0.008	0.033	0.033	0.960	0.006	0.025	0.026	0.960
		2. Naive	0.110	0.008	0.110	0.000	0.110	0.006	0.110	0.000
		3. 2SLS	0.008	0.058	0.058	0.900	0.013	0.039	0.041	0.855
		4. 2SRI	0.032	0.028	0.042	0.640	0.032	0.019	0.037	0.460
		5. Infeasible	0.000	0.032	0.032	0.965	0.001	0.024	0.024	0.965
	(b)	1. Proposed	0.007	0.034	0.035	0.985	0.004	0.027	0.028	0.990
		2. Naive	0.114	0.008	0.114	0.000	0.115	0.005	0.115	0.000
		3. 2SLS	−0.172	2.463	2.469	0.900	−0.010	0.428	0.428	0.915
		4. 2SRI	0.030	0.025	0.039	0.675	0.031	0.019	0.037	0.480
		5. Infeasible	0.000	0.032	0.032	0.955	0.000	0.025	0.025	0.950
	(c)	1. Proposed	0.008	0.046	0.047	0.975	0.005	0.034	0.034	0.950
		2. Naive	0.128	0.009	0.129	0.000	0.128	0.006	0.129	0.000
		3. 2SLS	0.008	0.063	0.063	0.885	0.011	0.036	0.037	0.880
		4. 2SRI	0.057	0.067	0.088	0.660	0.059	0.041	0.072	0.470
		5. Infeasible	0.000	0.044	0.044	0.930	0.002	0.029	0.029	0.955
	(d)	1. Proposed	0.008	0.045	0.046	0.970	0.004	0.038	0.038	0.960
		2. Naive	0.134	0.009	0.134	0.000	0.134	0.006	0.134	0.000
		3. 2SLS	0.059	0.594	0.597	0.865	0.070	0.783	0.787	0.855
		4. 2SRI	0.051	0.056	0.076	0.710	0.059	0.038	0.070	0.470
		5. Infeasible	−0.003	0.042	0.042	0.955	−0.003	0.028	0.028	0.950
Greatly misspecified	(a)	1. Proposed	0.017	0.027	0.032	0.915	0.018	0.022	0.028	0.895
		2. Naive	0.102	0.008	0.102	0.000	0.101	0.005	0.102	0.000
		3. 2SLS	0.007	0.060	0.060	0.915	0.014	0.039	0.042	0.860
		4. 2SRI	0.034	0.027	0.044	0.630	0.035	0.019	0.040	0.430
		5. Infeasible	0.001	0.022	0.023	0.955	0.000	0.017	0.017	0.950
	(b)	1. Proposed	0.018	0.029	0.034	0.905	0.018	0.022	0.028	0.915
		2. Naive	0.105	0.008	0.105	0.000	0.105	0.005	0.105	0.000
		3. 2SLS	0.114	1.648	1.652	0.905	0.014	0.568	0.568	0.910
		4. 2SRI	0.033	0.024	0.041	0.635	0.035	0.019	0.039	0.445
		5. Infeasible	0.001	0.022	0.022	0.955	0.000	0.017	0.017	0.965
	(c)	1. Proposed	0.024	0.033	0.041	0.870	0.023	0.025	0.034	0.865
		2. Naive	0.115	0.008	0.115	0.000	0.115	0.006	0.115	0.000
		3. 2SLS	0.010	0.065	0.065	0.895	0.013	0.035	0.038	0.895
		4. 2SRI	0.064	0.061	0.088	0.635	0.064	0.039	0.075	0.455
		5. Infeasible	0.001	0.025	0.025	0.940	0.001	0.017	0.017	0.965
	(d)	1. Proposed	0.024	0.034	0.042	0.875	0.025	0.026	0.036	0.860
		2. Naive	0.118	0.009	0.119	0.000	0.118	0.007	0.118	0.000
		3. 2SLS	0.033	1.832	1.832	0.875	0.054	0.568	0.570	0.840
		4. 2SRI	0.055	0.061	0.082	0.705	0.063	0.037	0.073	0.430
		5. Infeasible	−0.003	0.025	0.025	0.975	−0.002	0.017	0.017	0.940

(a): Strong common predictor and strong confounder; (b): Weak common predictor and strong confounder; (c): Strong common predictor and weak confounder; (d): Weak common predictor and weak confounder.

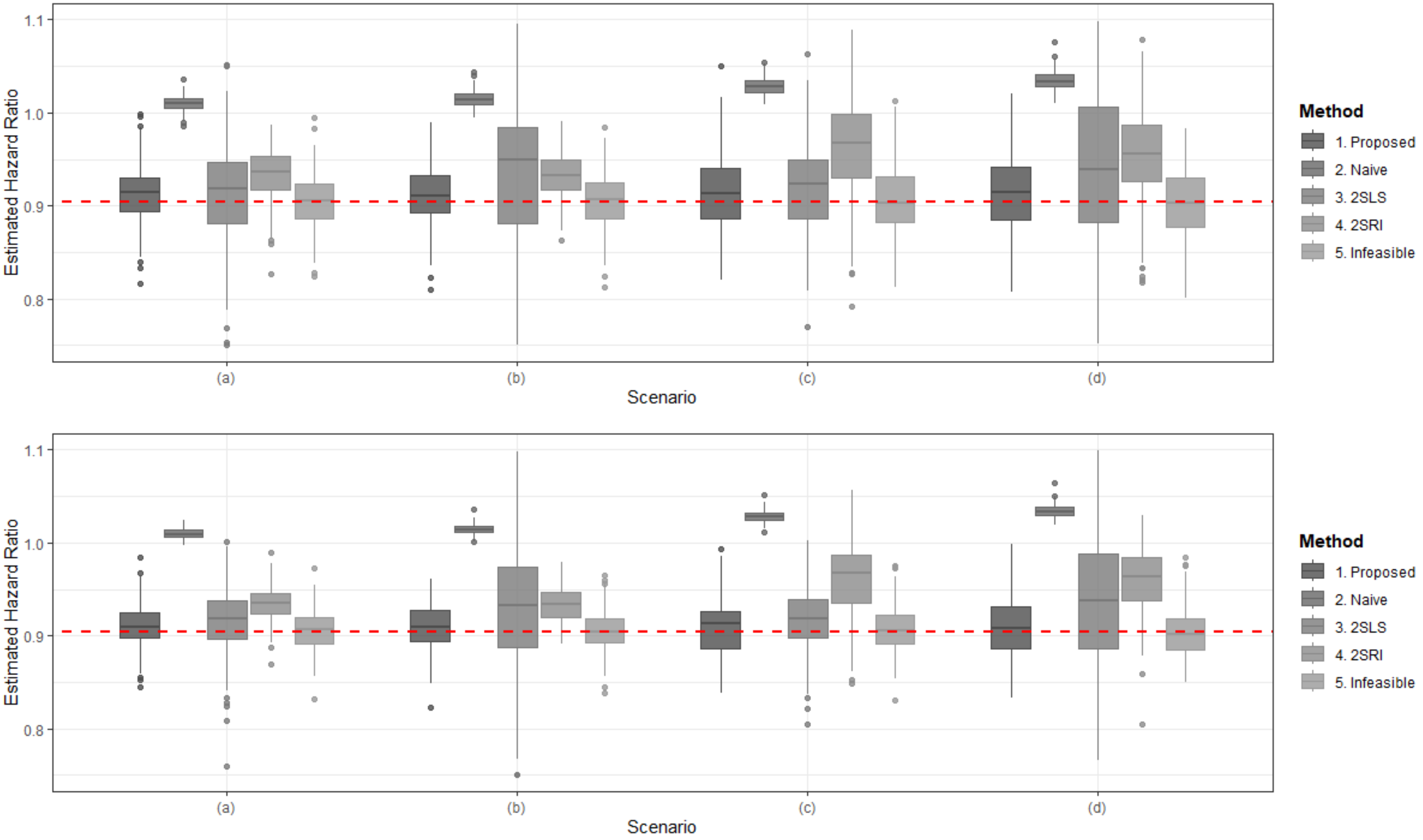

Figure B.10: Box plots of hazard ratio estimates for each method under additional scenario 1 (Slightly misspecified): The iteration time is 200. The true values of hazard ratio is $\exp\{-0.1\} \approx 0.905$.

Upper figure: the sample is 600; lower figure: the sample is 1200;
(a): Strong common predictor and strong confounder; (b): Weak common predictor and strong confounder;
(c): Strong common predictor and weak confounder; (d): Weak common predictor and weak confounder.

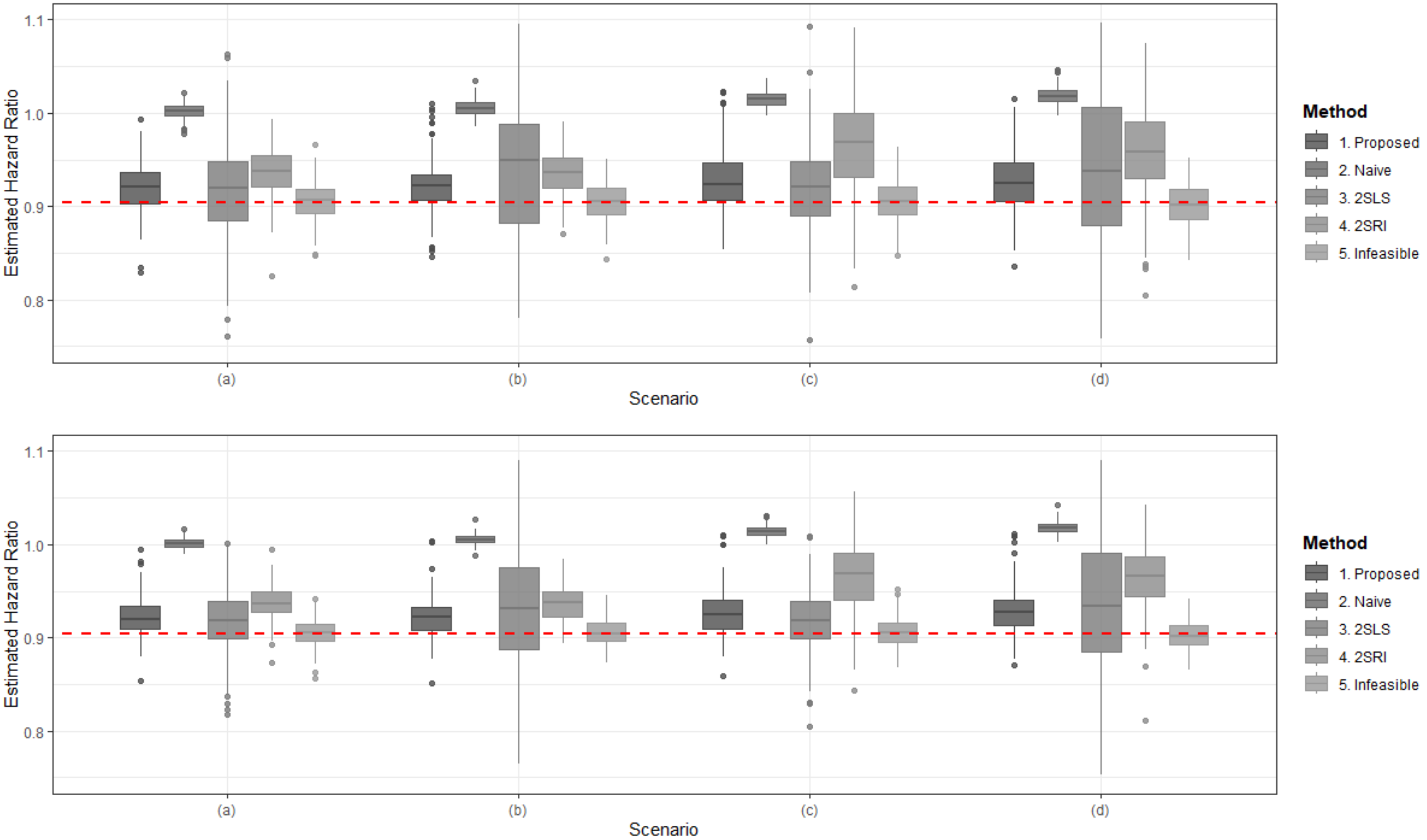

Figure B.11: Box plots of hazard ratio estimates for each method under additional scenario 1 (Greatly misspecified): The iteration time is 200. The true values of hazard ratio is $\exp\{-0.1\} \approx 0.905$.

Upper figure: the sample is 600; lower figure: the sample is 1200;

(a): Strong common predictor and strong confounder; (b): Weak common predictor and strong confounder;

(c): Strong common predictor and weak confounder; (d): Weak common predictor and weak confounder.

B.5.2 Additional scenario 2: True likelihood for exposure variable A is normal; analysis model is log-normal

We consider a normal distribution for the treatment variable (i.e., exactly as in the main manuscript), but apply a non-normal likelihood. Specifically, we use a log-normal distribution as the likelihood. To set up this scenario, we use the following transformation: assuming that a variable X follows a normal distribution, then $Y = \exp\{X\}$ follows a log-normal distribution. Based on this relationship, we apply $\log(A)$, enabling us to use the same estimation procedure as in the main manuscript, since in this scenario, we assume that the treatment variable A follows a log-normal distribution.

The results are summarized in Table B.4 and Figures B.12. In this scenario, likelihood misspecification can lead to non-negligible bias in the estimated treatment effect β_a, and this bias does not depend on the strength of the common predictor (i.e., IV). Specifically, the results are similar to those of the naive method, indicating that our proposed method cannot adequately adjust for the effects of unmeasured confounders. However, note that other methods yield more biased or unstable results compared to these two methods; in terms of RMSE, both the proposed and naive methods are preferable.

From the results of the above two additional scenarios, we conclude that using the normal distribution as the likelihood for the treatment variable is a reasonable initial choice. Additionally, when the treatment variable exhibits skewness, it may be useful to consider more flexible likelihood functions, such as the log-normal distribution. Even in such cases, we think that applying our proposed method remains valuable, as adjusting for confounders can potentially provide more accurate results than the naive approach.

Table B.4: Summary of Hazard Ratio Estimates under additional scenario 2: The iteration time is 200, and the true values of the log hazard ratio is −0.1. Bias, empirical standard error (ESE), root mean squared error (RMSE), and coverage probability (CP) of the estimated log-hazard ratio in 200 iterations by estimation methods ("Method" column) are summarized.

Scenario	Method	log–hazard ratio							
		$n = 600$				$n = 1200$			
		**Bias**	**ESE**	**RMSE**	**CP**	**Bias**	**ESE**	**RMSE**	**CP**
(a)	1. Proposed	0.113	0.008	0.113	0.000	0.115	0.007	0.116	0.000
	2. Naive	0.111	0.008	0.111	0.000	0.110	0.006	0.111	0.000
	3. 2SLS	−0.936	0.640	1.134	0.550	−0.906	0.387	0.985	0.295
	4. 2SRI	0.193	0.023	0.194	0.000	0.191	0.016	0.191	0.000
	5. Infeasible	−0.001	0.041	0.041	0.950	−0.001	0.030	0.030	0.960
(b)	1. Proposed	0.122	0.009	0.122	0.000	0.124	0.007	0.124	0.000
	2. Naive	0.116	0.008	0.117	0.000	0.116	0.005	0.116	0.000
	3. 2SLS	0.138	9.166	9.167	0.885	−1.356	5.939	6.092	0.845
	4. 2SRI	0.202	0.022	0.203	0.000	0.198	0.016	0.198	0.000
	5. Infeasible	0.003	0.041	0.041	0.940	0.001	0.028	0.028	0.960
(c)	1. Proposed	0.135	0.009	0.135	0.000	0.137	0.008	0.138	0.000
	2. Naive	0.131	0.009	0.131	0.000	0.131	0.006	0.131	0.000
	3. 2SLS	−0.831	0.481	0.960	0.510	−0.837	0.351	0.907	0.280
	4. 2SRI	0.264	0.028	0.266	0.000	0.262	0.022	0.263	0.000
	5. Infeasible	−0.005	0.052	0.052	0.950	−0.001	0.032	0.032	0.975
(d)	1. Proposed	0.143	0.011	0.144	0.000	0.148	0.009	0.148	0.000
	2. Naive	0.137	0.009	0.137	0.000	0.138	0.006	0.138	0.000
	3. 2SLS	−0.415	4.812	4.829	0.890	−0.872	7.220	7.272	0.860
	4. 2SRI	0.278	0.026	0.279	0.000	0.276	0.023	0.277	0.000
	5. Infeasible	0.001	0.055	0.055	0.945	0.003	0.039	0.040	0.940

(a): Strong common predictor and strong confounder; (b): Weak common predictor and strong confounder; (c): Strong common predictor and weak confounder; (d): Weak common predictor and weak confounder.

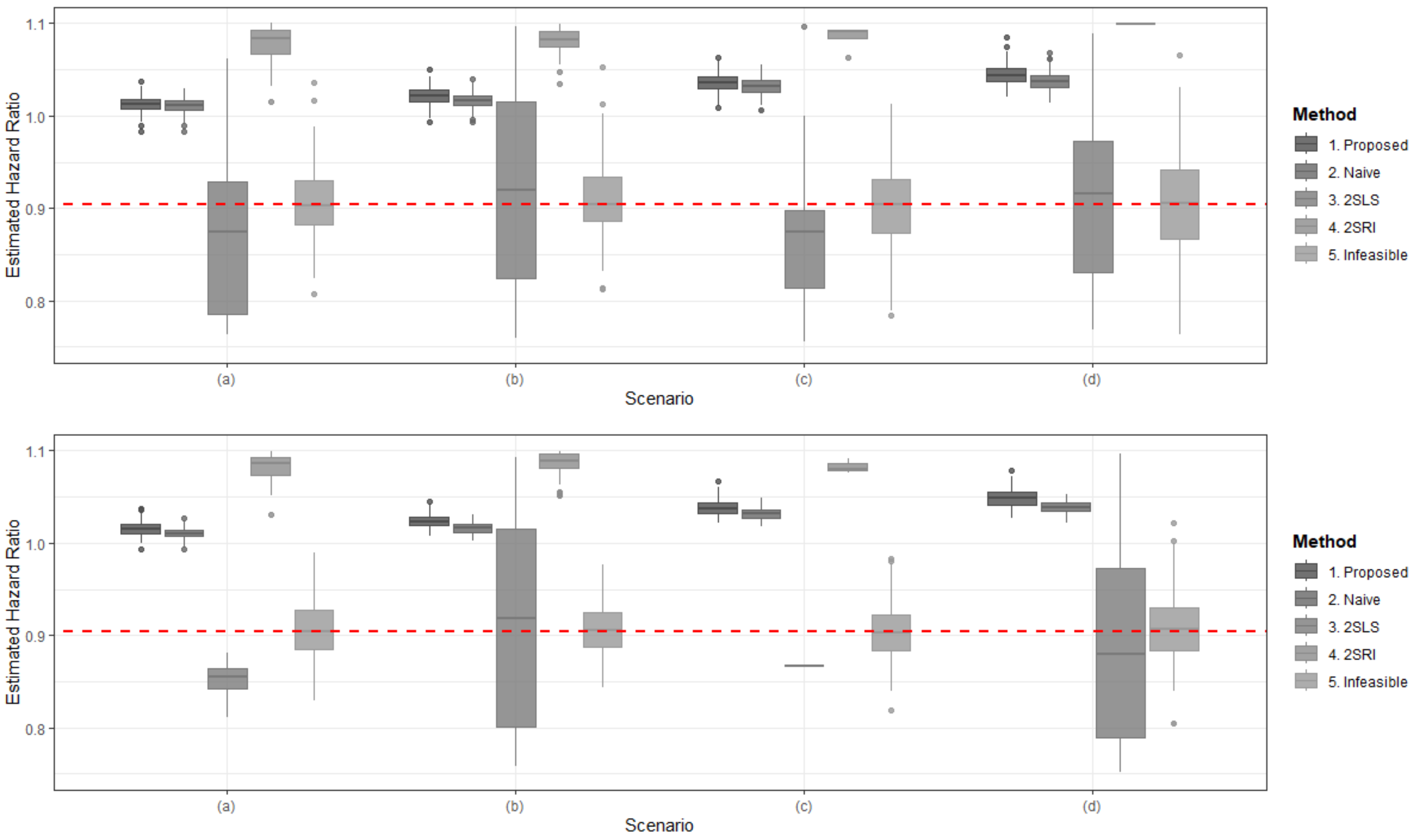

Figure B.12: Box plots of hazard ratio estimates for each method under additional scenario 2: The iteration time is 200. The true values of hazard ratio is $\exp\{-0.1\} \approx 0.905$.

Upper figure: the sample is 600; lower figure: the sample is 1200;

(a): Strong common predictor and strong confounder; (b): Weak common predictor and strong confounder;

(c): Strong common predictor and weak confounder; (d): Weak common predictor and weak confounder.

B.5.3 Additional scenario 3: Violation of the exclusion restriction assumption

We consider a scenario with a direct relationship from the IV to the outcome. Specifically, we add

- Subtle confounder: $0.1 \times z_i$
- Obvious confounder: $1 \times z_i$

to the outcome model described in Appendix B.1. As mentioned in the main manuscript and Appendix A.1, we use a shrinkage prior for β_z. We investigate how this prior affects the parameter of interest, β_a.

The results are summarized in Table B.5, and Figures B.13 and B.14. In the "Subtle confounder" setting, the proposed method performs well compared to the other scenarios. In the "Obvious confounder" setting, the proposed estimator is slightly drawn toward the direction of the "Naive" estimator. However, the resulting bias remains small relative to that of the other methods, and it diminishes as the sample size increases.

Therefore, depending on the simulation setting, we consider the specification of the shrinkage priors to be acceptable, or at least that their impact on the performance of our proposed method is limited.

Table B.5: Summary of Hazard Ratio Estimates under additional scenario 3: The iteration time is 200, and the true values of the log hazard ratio is −0.1. Bias, empirical standard error (ESE), root mean squared error (RMSE), and coverage probability (CP) of the estimated log-hazard ratio in 200 iterations by estimation methods ("Method" column) are summarized.

			**log–hazard ratio**							
**Setting**	**Scenario**	**Method**	$n = 600$				$n = 1200$			
			**Bias**	**ESE**	**RMSE**	**CP**	**Bias**	**ESE**	**RMSE**	**CP**
Subtle confounder	(a)	1. Proposed	0.007	0.035	0.036	0.965	0.005	0.028	0.028	0.980
		2. Naive	0.116	0.008	0.116	0.000	0.115	0.006	0.116	0.000
		3. 2SLS	0.070	0.050	0.086	0.540	0.072	0.030	0.078	0.310
		4. 2SRI	0.031	0.026	0.040	0.630	0.031	0.019	0.037	0.475
		5. Infeasible	0.000	0.041	0.041	0.955	0.000	0.030	0.030	0.960
	(b)	1. Proposed	0.007	0.036	0.037	0.970	0.005	0.027	0.028	0.970
		2. Naive	0.122	0.008	0.122	0.000	0.121	0.005	0.122	0.000
		3. 2SLS	0.284	1.859	1.881	0.640	0.235	1.186	1.209	0.405
		4. 2SRI	0.030	0.026	0.040	0.655	0.031	0.018	0.036	0.465
		5. Infeasible	0.004	0.041	0.042	0.950	0.001	0.029	0.029	0.965
	(c)	1. Proposed	0.003	0.048	0.048	0.975	0.004	0.035	0.035	0.970
		2. Naive	0.138	0.009	0.138	0.000	0.137	0.006	0.137	0.000
		3. 2SLS	0.072	0.042	0.083	0.570	0.071	0.031	0.077	0.340
		4. 2SRI	0.055	0.054	0.078	0.665	0.057	0.036	0.068	0.530
		5. Infeasible	−0.005	0.052	0.052	0.960	−0.001	0.033	0.033	0.970
	(d)	1. Proposed	0.004	0.053	0.054	0.980	0.004	0.039	0.039	0.970
		2. Naive	0.144	0.009	0.144	0.000	0.144	0.006	0.144	0.000
		3. 2SLS	0.376	2.755	2.781	0.610	0.156	0.428	0.455	0.420
		4. 2SRI	0.055	0.056	0.078	0.615	0.057	0.039	0.069	0.505
		5. Infeasible	0.000	0.055	0.055	0.950	0.003	0.039	0.039	0.940
Obvious confounder	(a)	1. Proposed	0.010	0.046	0.047	0.955	0.001	0.025	0.025	0.980
		2. Naive	0.168	0.009	0.168	0.000	0.167	0.006	0.167	0.000
		3. 2SLS	0.475	0.111	0.488	0.000	0.472	0.073	0.477	0.000
		4. 2SRI	0.037	0.029	0.047	0.550	0.038	0.021	0.043	0.320
		5. Infeasible	−0.002	0.038	0.038	0.965	−0.001	0.027	0.027	0.955
	(b)	1. Proposed	0.019	0.052	0.055	0.935	0.005	0.030	0.030	0.975
		2. Naive	0.173	0.009	0.173	0.000	0.172	0.006	0.172	0.000
		3. 2SLS	1.489	15.868	15.938	0.000	1.820	7.504	7.722	0.000
		4. 2SRI	0.036	0.032	0.048	0.505	0.038	0.021	0.044	0.340
		5. Infeasible	0.004	0.040	0.040	0.950	0.001	0.028	0.028	0.940
	(c)	1. Proposed	0.012	0.061	0.063	0.955	0.002	0.041	0.041	0.975
		2. Naive	0.196	0.010	0.196	0.000	0.195	0.007	0.195	0.000
		3. 2SLS	0.455	0.086	0.463	0.000	0.450	0.055	0.454	0.000
		4. 2SRI	0.060	0.062	0.087	0.615	0.061	0.042	0.074	0.510
		5. Infeasible	−0.006	0.050	0.051	0.935	−0.001	0.032	0.032	0.975
	(d)	1. Proposed	0.020	0.071	0.074	0.920	0.005	0.049	0.049	0.950
		2. Naive	0.201	0.011	0.202	0.000	0.201	0.008	0.201	0.000
		3. 2SLS	2.916	21.919	22.113	0.000	2.063	9.213	9.441	0.000
		4. 2SRI	0.059	0.063	0.086	0.585	0.063	0.045	0.077	0.410
		5. Infeasible	0.000	0.053	0.053	0.950	0.002	0.036	0.036	0.950

(a): Strong common predictor and strong confounder; (b): Weak common predictor and strong confounder; (c): Strong common predictor and weak confounder; (d): Weak common predictor and weak confounder.

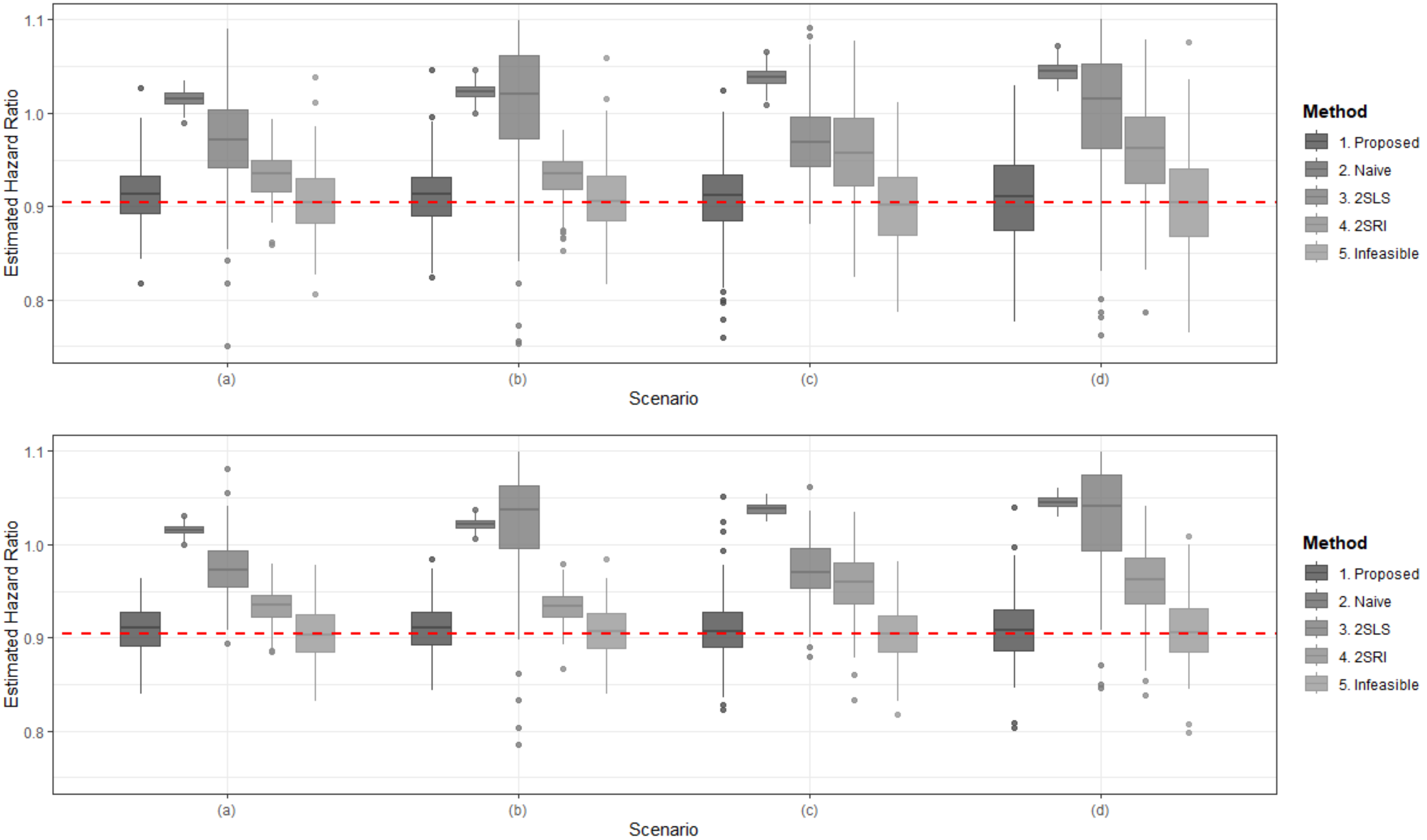

Figure B.13: Box plots of hazard ratio estimates for each method under additional scenario 3 (Subtle confounder): The iteration time is 200. The true values of hazard ratio is $\exp\{-0.1\} \approx 0.905$.

Upper figure: the sample is 600; lower figure: the sample is 1200;
(a): Strong common predictor and strong confounder; (b): Weak common predictor and strong confounder;
(c): Strong common predictor and weak confounder; (d): Weak common predictor and weak confounder.

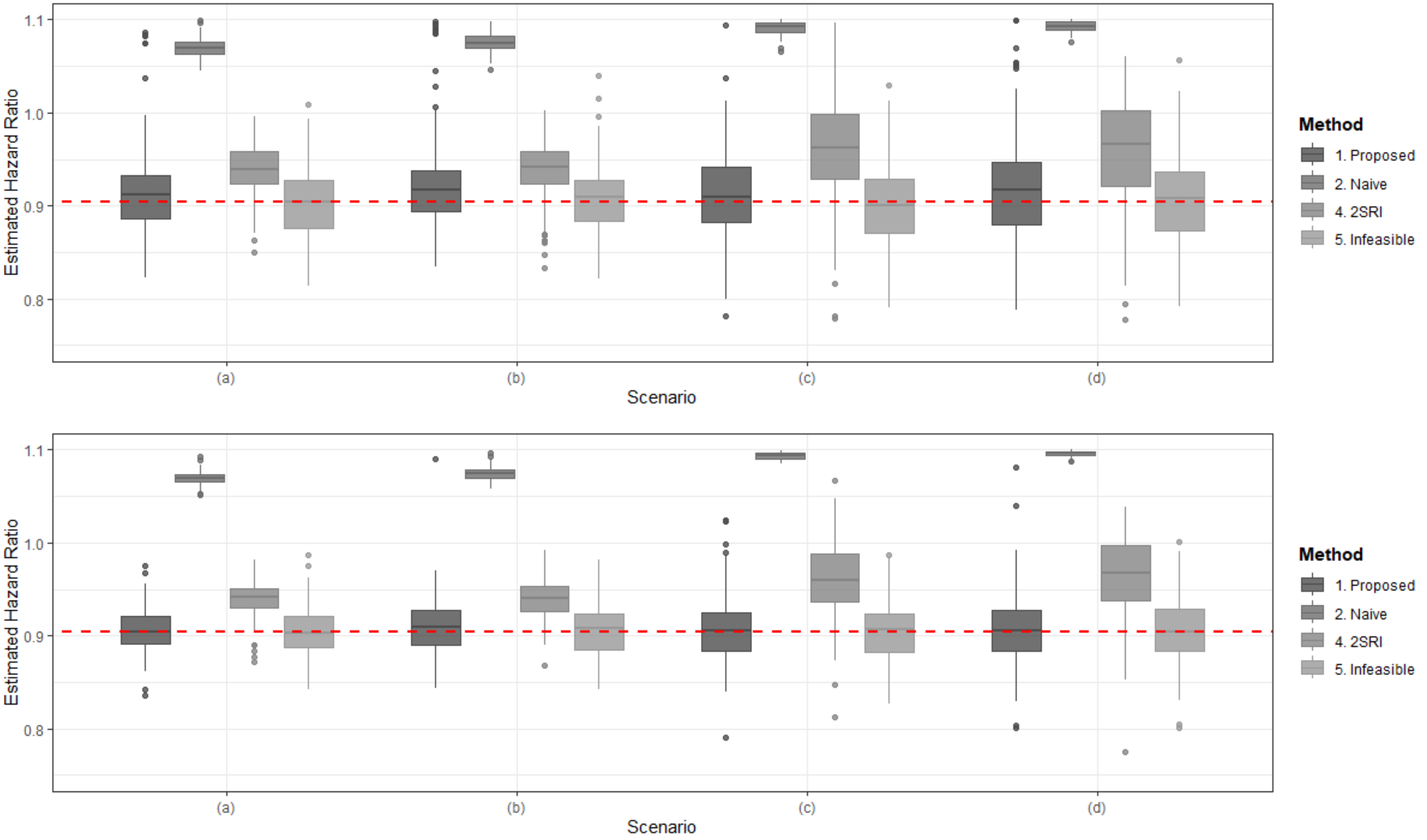

Figure B.14: Box plots of hazard ratio estimates for each method under additional scenario 3 (Obvious confounder): The iteration time is 200. The true values of hazard ratio is $\exp\{-0.1\} \approx 0.905$.

Upper figure: the sample is 600; lower figure: the sample is 1200;
Due to the substantial bias of the 2SLS estimator, its boxplots are omitted;
(a): Strong common predictor and strong confounder; (b): Weak common predictor and strong confounder;
(c): Strong common predictor and weak confounder; (d): Weak common predictor and weak confounder.

B.5.4 Additional scenario 4: Continuous unmeasured confounders

We consider a situation where the unmeasured confounder follows a continuous distribution. This is motivated by the fact that, as mentioned in the main manuscript, our proposed method strongly relies on a data structure that assumes an underlying cluster structure, which implicitly suggests that the unmeasured confounder has discrete support.

Specifically, we consider the following data-generating mechanisms in this section. Except for the modifications described below, all other settings are the same as those used in the main simulation experiments. The unmeasured confounder U_i follows a multivariate normal distribution:

$$U_i = \begin{pmatrix} U_{1i} \\ U_{2i} \\ U_{3i} \end{pmatrix} \overset{i.i.d.}{\sim} N_3 \left(\mathbf{0}, \begin{pmatrix} 1 & 0 & \rho \\ 0 & 1 & \rho \\ \rho & \rho & 1 \end{pmatrix} \right),$$

where

- Weak unmeasured confounder: $\rho = 0.3$
- Strong unmeasured confounder: $\rho = 0.6$.

Using U_i, we define the following models. For the treatment model,

$$A \mid z_i, v_i, k \sim N\left(\alpha_{0i} + z_i\alpha_z + v_i\alpha_{vi}, 0.5^2\right),$$

where $\alpha_{0i} = 16 + 0.4 \times U_{1i}$ and $\alpha_{vi} = 4 + 0.2 \times U_{2i}$. For the outcome model,

$$\lambda(t_i \mid a_i, v_i, k) = \lambda_{0i} \exp\left\{-0.1a_i + 0.1v_i\right\},$$

where $\lambda_{0i} = 0.1 \times \exp\{0.4 \times U_{3i}\}$.

The results are summarized in Table B.6, and Figures B.15 and B.16. For our proposed

method, a clear bias that is almost identical to that of the "Naive" estimator appears under the small sample setting. In the large sample setting, the bias is slightly reduced. These results suggest that when unmeasured confounders follow a continuous distribution, our proposed method cannot fully adjust for their effects.

The simulation results reinforce that the assumed data structure is a key assumption for the validity of our proposed method, as discussed in the main manuscript. However, since unmeasured confounders are typically unobservable, assuming a clustered structure is just one possible approach. Therefore, using at least one alternative method, such as 2SLS, as a form of sensitivity analysis is a prudent strategy for interpreting the results.

Table B.6: Summary of Hazard Ratio Estimates under additional scenario 4: The iteration time is 200, and the true values of the log hazard ratio is −0.1. Bias, empirical standard error (ESE), root mean squared error (RMSE), and coverage probability (CP) of the estimated log-hazard ratio in 200 iterations by estimation methods ("Method" column) are summarized.

Setting	Scenario	Method	log–hazard ratio							
			$n = 600$				$n = 1200$			
			**Bias**	**ESE**	**RMSE**	**CP**	**Bias**	**ESE**	**RMSE**	**CP**
Weak unmeasured confounder	(a)	1. Proposed	0.095	0.050	0.107	0.790	0.084	0.035	0.091	0.595
		2. Naive	0.096	0.059	0.112	0.665	0.096	0.042	0.105	0.370
		3. 2SLS	0.001	0.050	0.050	0.940	0.004	0.032	0.032	0.950
		4. 2SRI	0.029	0.018	0.034	0.630	0.029	0.012	0.031	0.350
		5. Infeasible	−0.007	0.057	0.058	0.955	−0.005	0.043	0.043	0.945
	(b)	1. Proposed	0.095	0.054	0.109	0.800	0.084	0.037	0.092	0.580
		2. Naive	0.090	0.061	0.109	0.670	0.092	0.044	0.102	0.395
		3. 2SLS	−0.031	0.272	0.274	0.915	−0.038	0.360	0.362	0.945
		4. 2SRI	0.028	0.016	0.032	0.610	0.030	0.012	0.032	0.320
		5. Infeasible	−0.012	0.062	0.063	0.925	−0.009	0.045	0.045	0.925
	(c)	1. Proposed	0.095	0.052	0.108	0.810	0.081	0.032	0.087	0.720
		2. Naive	0.090	0.060	0.108	0.645	0.085	0.038	0.093	0.460
		3. 2SLS	0.002	0.044	0.044	0.975	0.003	0.032	0.032	0.935
		4. 2SRI	0.052	0.037	0.064	0.655	0.054	0.023	0.059	0.395
		5. Infeasible	−0.001	0.061	0.061	0.930	−0.005	0.041	0.041	0.950
	(d)	1. Proposed	0.096	0.052	0.109	0.835	0.089	0.034	0.095	0.540
		2. Naive	0.085	0.057	0.102	0.685	0.091	0.040	0.099	0.385
		3. 2SLS	−0.009	0.170	0.170	0.905	0.001	0.102	0.102	0.940
		4. 2SRI	0.054	0.033	0.063	0.630	0.055	0.024	0.060	0.325
		5. Infeasible	−0.005	0.059	0.059	0.935	0.002	0.042	0.042	0.955
Strong unmeasured confounder	(a)	1. Proposed	0.185	0.063	0.195	0.270	0.171	0.042	0.176	0.025
		2. Naive	0.192	0.059	0.201	0.085	0.190	0.042	0.195	0.010
		3. 2SLS	0.001	0.051	0.051	0.930	0.004	0.033	0.033	0.945
		4. 2SRI	0.029	0.018	0.035	0.585	0.029	0.013	0.031	0.375
		5. Infeasible	−0.007	0.061	0.062	0.950	−0.006	0.047	0.048	0.950
	(b)	1. Proposed	0.184	0.063	0.194	0.240	0.170	0.042	0.175	0.035
		2. Naive	0.181	0.060	0.191	0.100	0.184	0.043	0.189	0.010
		3. 2SLS	−0.029	0.275	0.277	0.910	−0.038	0.353	0.355	0.930
		4. 2SRI	0.028	0.016	0.033	0.620	0.030	0.012	0.032	0.315
		5. Infeasible	−0.014	0.066	0.068	0.930	−0.009	0.046	0.047	0.945
	(c)	1. Proposed	0.173	0.061	0.184	0.310	0.154	0.037	0.158	0.045
		2. Naive	0.174	0.060	0.184	0.160	0.168	0.039	0.172	0.005
		3. 2SLS	0.001	0.044	0.044	0.975	0.002	0.033	0.033	0.940
		4. 2SRI	0.052	0.036	0.063	0.660	0.053	0.023	0.058	0.405
		5. Infeasible	0.001	0.065	0.065	0.920	−0.005	0.044	0.044	0.950
	(d)	1. Proposed	0.173	0.057	0.182	0.285	0.161	0.039	0.166	0.035
		2. Naive	0.165	0.058	0.175	0.170	0.172	0.042	0.177	0.015
		3. 2SLS	−0.011	0.172	0.172	0.915	0.000	0.104	0.104	0.940
		4. 2SRI	0.054	0.033	0.064	0.630	0.056	0.024	0.061	0.320
		5. Infeasible	−0.006	0.062	0.063	0.940	0.002	0.046	0.046	0.935

(a): Strong common predictor and strong confounder; (b): Weak common predictor and strong confounder; (c): Strong common predictor and weak confounder; (d): Weak common predictor and weak confounder.

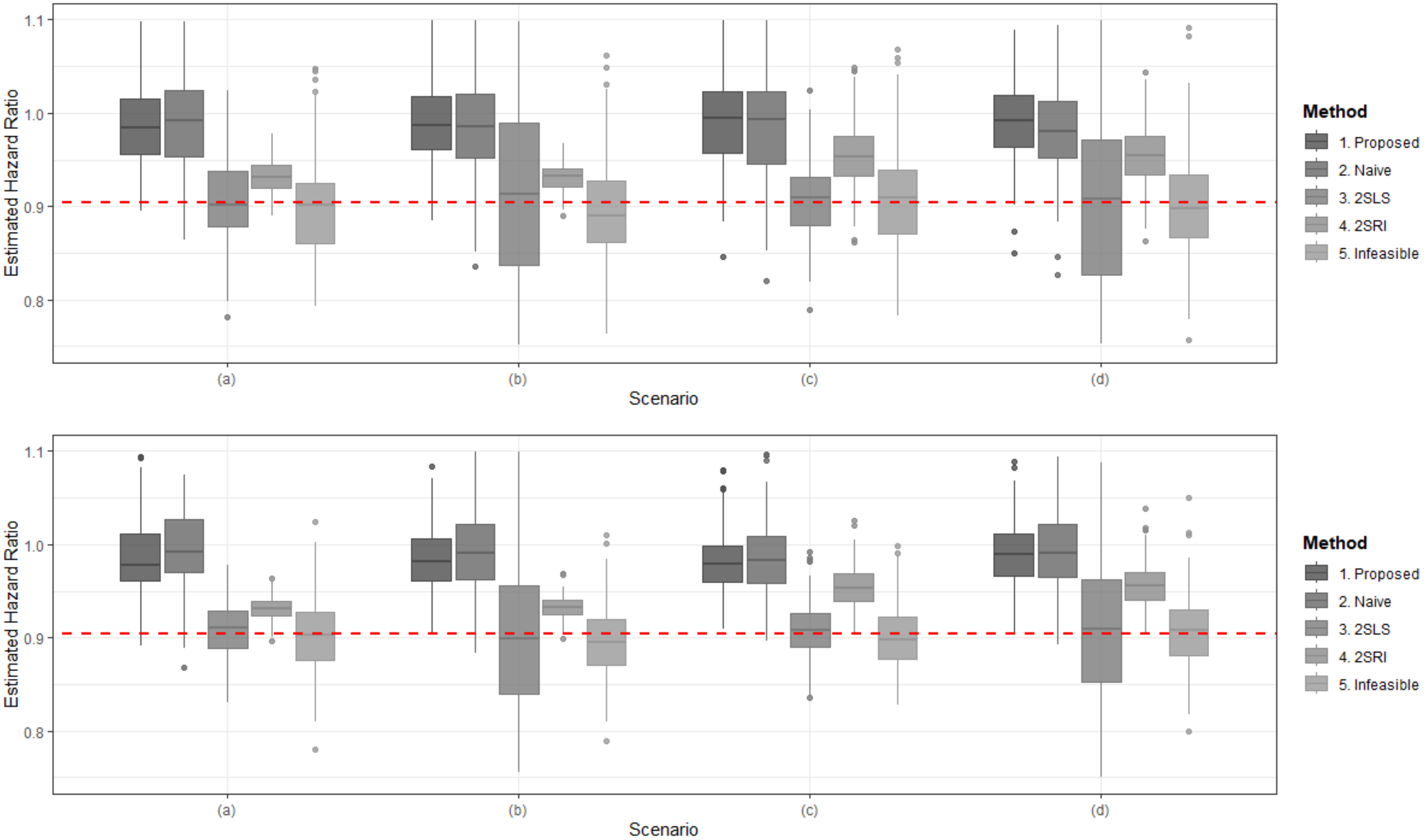

Figure B.15: Box plots of hazard ratio estimates for each method under additional scenario 4 (Weak unmeasured confounder): The iteration time is 200. The true values of hazard ratio is $\exp\{-0.1\} \approx 0.905$.

Upper figure: the sample is 600; lower figure: the sample is 1200;
(a): Strong common predictor and strong confounder; (b): Weak common predictor and strong confounder;
(c): Strong common predictor and weak confounder; (d): Weak common predictor and weak confounder.

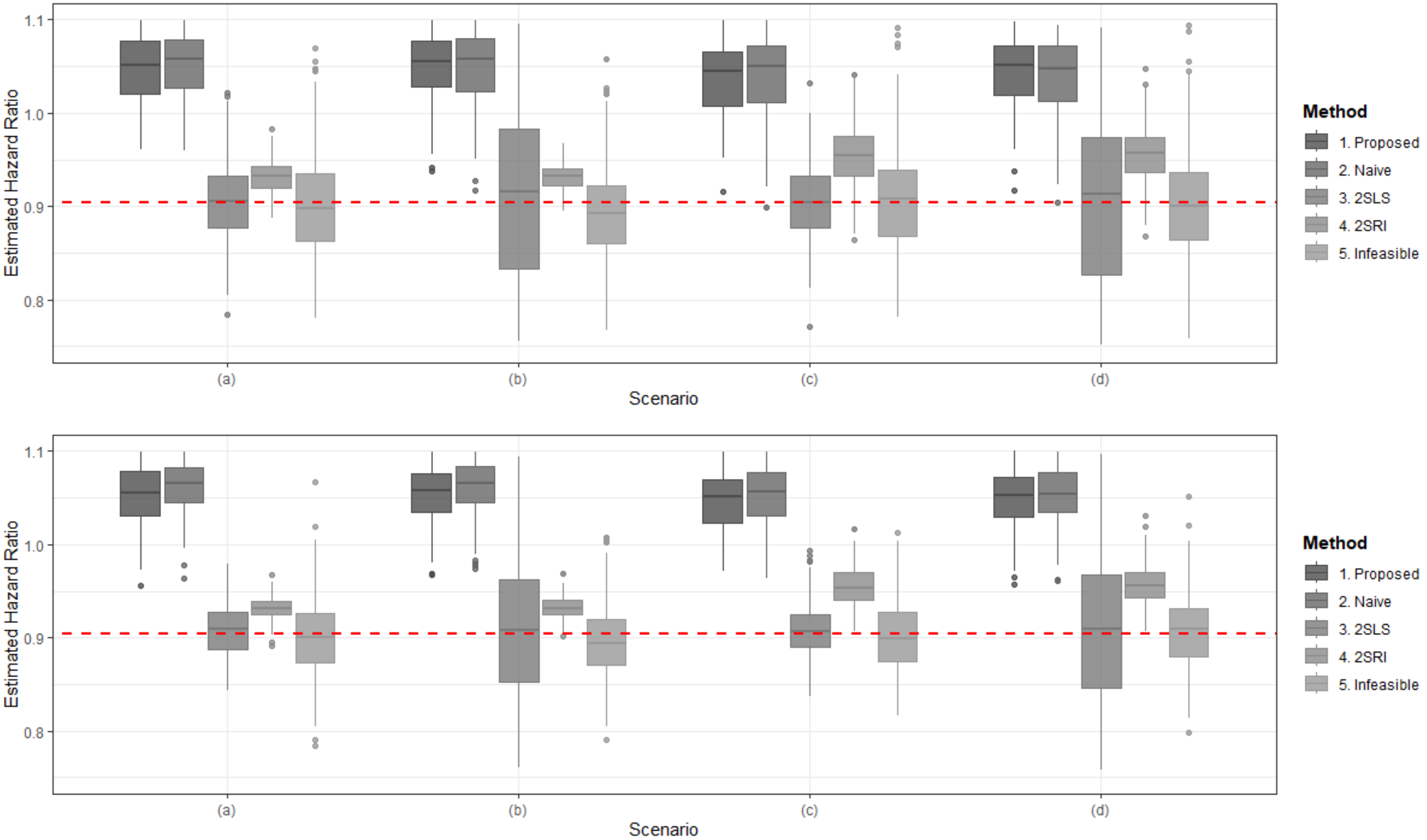

Figure B.16: Box plots of hazard ratio estimates for each method under additional scenario 4 (Strong unmeasured confounder): The iteration time is 200. The true values of hazard ratio is $\exp\{-0.1\} \approx 0.905$.

Upper figure: the sample is 600; lower figure: the sample is 1200;
(a): Strong common predictor and strong confounder; (b): Weak common predictor and strong confounder;
(c): Strong common predictor and weak confounder; (d): Weak common predictor and weak confounder.

B.5.5 Additional scenario 5: No cluster effects

We assume there are no cluster effects. In other words, there are no unmeasured confounders, and the measured confounder $\boldsymbol{x}$ is sufficient. In this scenario, all subjects are assigned to cluster ID: 1 (i.e., $K_i \equiv 1$). Under this condition, our proposed method includes redundant parameters. We investigate how including these affects parameter estimation.

The results are summarized in Table B.7 and Figures B.17. Note that in this scenario, the "Naive" and "Infeasible" methods yield identical, well-performing results. Additionally, in situations (a) and (c), 2SLS performs well. Our proposed method also performs comparably, especially relative to "Infeasible." One reason is that, as discussed in Remark 1 of the main manuscript, our method leverages information from the treatment model, which may help improve the efficiency of estimating β_a. In any case, including redundant parameters does not adversely affect the estimates of the parameter of interest.

Table B.7: Summary of Hazard Ratio Estimates under additional scenario 5: The iteration time is 200, and the true values of the log hazard ratio is −0.1. Bias, empirical standard error (ESE), root mean squared error (RMSE), and coverage probability (CP) of the estimated log-hazard ratio in 200 iterations by estimation methods ("Method" column) are summarized.

		**log–hazard ratio**							
**Scenario**	**Method**	$n = 600$				$n = 1200$			
		**Bias**	**ESE**	**RMSE**	**CP**	**Bias**	**ESE**	**RMSE**	**CP**
(a)	1. Proposed	0.004	0.044	0.044	0.985	0.000	0.035	0.035	0.985
	2. Naive	−0.002	0.064	0.064	0.945	−0.005	0.046	0.047	0.940
	3. 2SLS	−0.003	0.046	0.046	0.930	−0.002	0.033	0.033	0.930
	4. 2SRI	0.024	0.016	0.029	0.680	0.025	0.010	0.027	0.365
	5. Infeasible	−0.002	0.064	0.064	0.945	−0.005	0.046	0.047	0.940
(b)	1. Proposed	0.005	0.047	0.047	0.995	0.002	0.035	0.035	0.985
	2. Naive	−0.001	0.062	0.062	0.965	−0.003	0.043	0.044	0.950
	3. 2SLS	−0.027	0.362	0.363	0.940	−0.005	0.110	0.110	0.915
	4. 2SRI	0.027	0.015	0.031	0.605	0.026	0.011	0.028	0.380
	5. Infeasible	−0.001	0.062	0.062	0.965	−0.003	0.043	0.044	0.950
(c)	1. Proposed	0.008	0.050	0.051	0.990	0.006	0.037	0.038	0.975
	2. Naive	0.003	0.064	0.064	0.960	0.002	0.044	0.044	0.960
	3. 2SLS	−0.001	0.045	0.045	0.945	−0.001	0.030	0.030	0.955
	4. 2SRI	0.050	0.029	0.058	0.660	0.050	0.023	0.055	0.360
	5. Infeasible	0.003	0.064	0.064	0.960	0.002	0.044	0.044	0.960
(d)	1. Proposed	0.006	0.052	0.053	0.975	0.006	0.036	0.037	0.965
	2. Naive	0.000	0.060	0.060	0.955	0.002	0.041	0.041	0.950
	3. 2SLS	−0.008	0.140	0.140	0.950	−0.009	0.088	0.088	0.950
	4. 2SRI	0.048	0.031	0.057	0.640	0.048	0.021	0.053	0.415
	5. Infeasible	0.000	0.060	0.060	0.955	0.002	0.041	0.041	0.950

(a): Strong common predictor and strong confounder; (b): Weak common predictor and strong confounder; (c): Strong common predictor and weak confounder; (d): Weak common predictor and weak confounder.

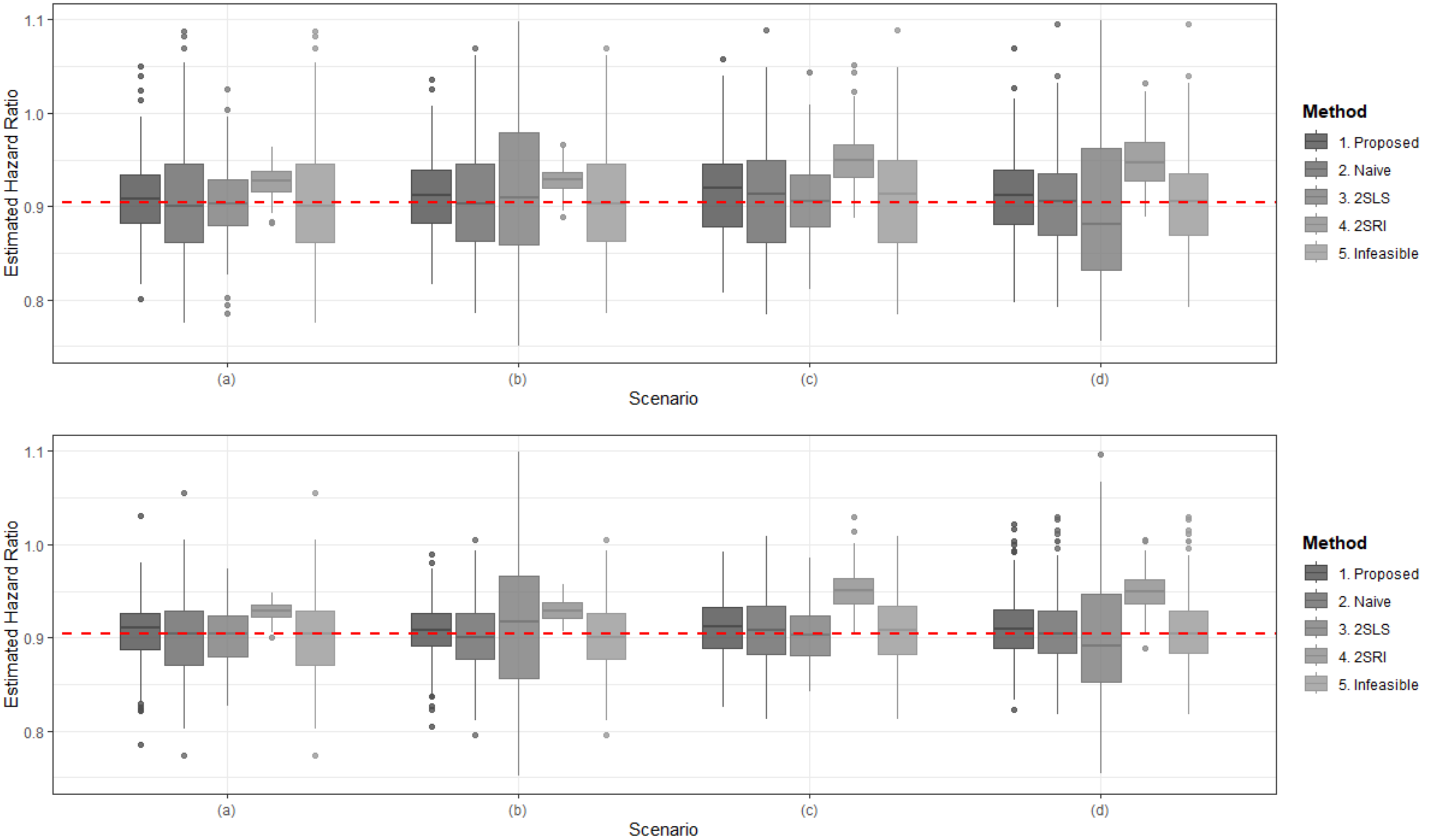

Figure B.17: Box plots of hazard ratio estimates for each method under additional scenario 5: The iteration time is 200. The true values of hazard ratio is $\exp\{-0.1\} \approx 0.905$.

Upper figure: the sample is 600; lower figure: the sample is 1200;

(a): Strong common predictor and strong confounder; (b): Weak common predictor and strong confounder;

(c): Strong common predictor and weak confounder; (d): Weak common predictor and weak confounder.

C Additional Information for UK Biobank Data Analysis

C.1 Likelihoods and prior distributions

In this data analysis, we consider the following models:

$$A_i = \alpha_{0i} + z_i\alpha_z + \boldsymbol{v}_i^\top \boldsymbol{\alpha}_v + \varepsilon_i, \quad \lambda_i(t) = \lambda_{0i}(t) \exp\left(a_i\beta_a + z_i\beta_z + \boldsymbol{v}_i^\top \boldsymbol{\beta}_v\right),$$

where $\varepsilon_i \overset{i.i.d.}{\sim} N(0, \sigma^2)$, A_i represents fruit intake, z_i is an allele score derived from 20 SNPs, and $\boldsymbol{v}_i$ includes age and sex.

For α_z and $\boldsymbol{\alpha}_v$, we adopt normal prior distributions: $N(0, 10^2)$. For σ, we assume an inverse gamma prior: $\text{InvGamma}(2, 2)$. For β_a and $\boldsymbol{\beta}_v$, we also use normal priors: $N(0, 2^2)$. For β_z, as described in Appendix A.1, we adopt the following shrinkage prior

$$\beta_z \mid \psi \sim N(0, 4 \times \psi^2), \quad \psi \sim C^+(0, 1).$$

Sampling for γ (see Appendix A.2), we assume a gamma prior: $\text{Gamma}(0.01, 100)$.

C.2 Additional analysis results

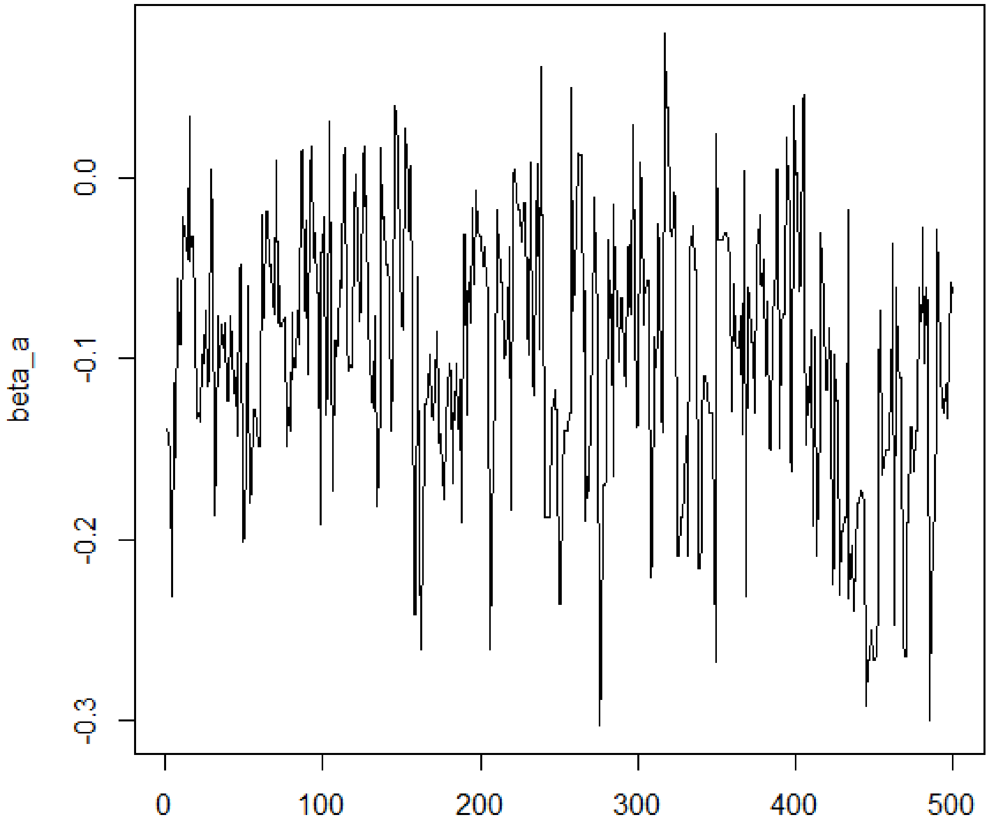

Figure C.18: Sampling plot of β_a for proposed procedure in UK Biobank dataset

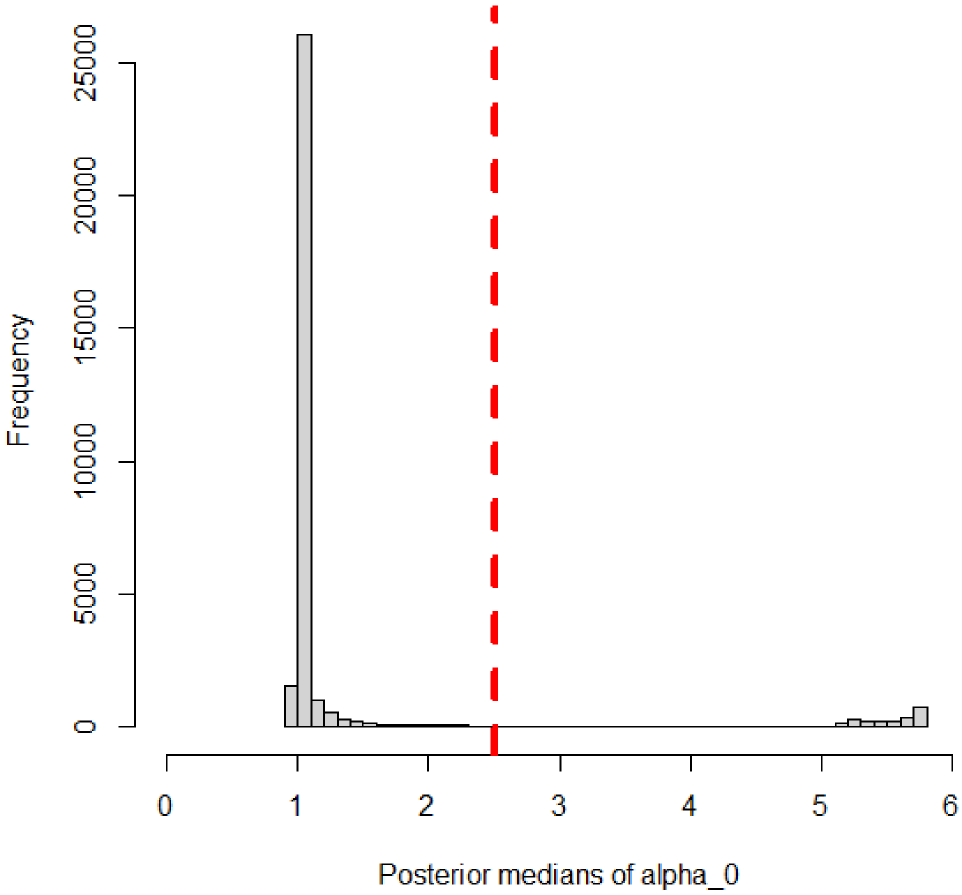

Figure C.19: Plot of α_{0k} for proposed procedure in UK Biobank dataset

Red dash line: cut off point of two groups;
Posterior medians ≥ 2.5: Health-conscious group; Posterior medians < 2.5: Health-unconscious group.

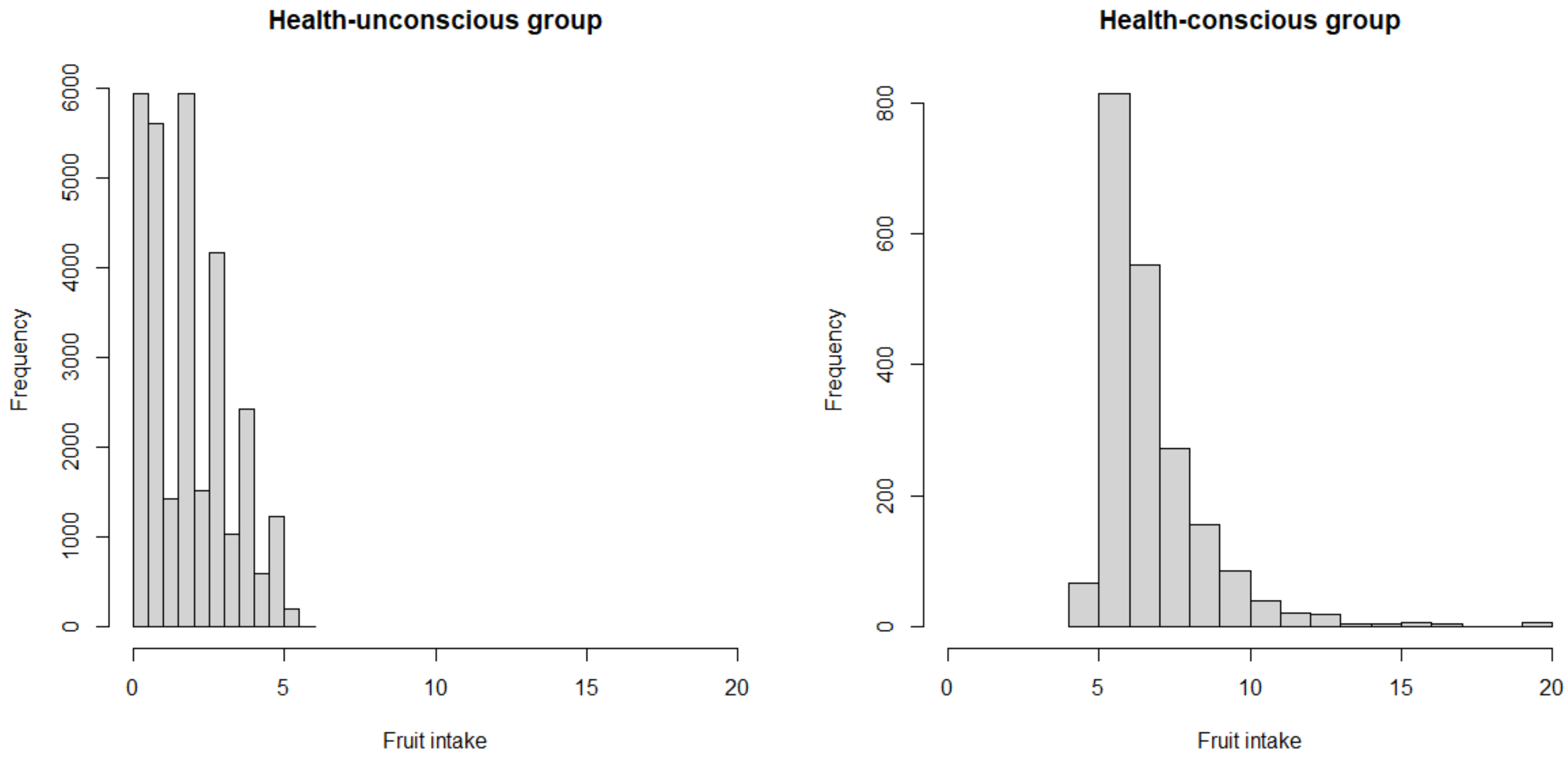

Figure C.20: Histogram of the exposure variable by groups in UK Biobank dataset

D Overcoming Homogeneous Treatment Effect Assumption and its Limitations

By modifying the model (2.2) in the main manuscript, we can consider a more complicated model that the parameter $\boldsymbol{\beta}$ may be different in each cluster:

$$\lambda_i(t) = \lambda_{0i}(t) \exp\left(\tilde{\boldsymbol{x}}_i^\top \boldsymbol{\beta}_i\right). \tag{D.1}$$

For this model, the posterior sampling algorithm described in Section 3.2 needs to be slightly modified, however, sampling for $\boldsymbol{\beta}_i$ can be considered in the same manner. In (D.1), the treatment effect β_{ai} may change for each (unknown) cluster; i.e., unmeasured confounders. In other words, (D.1) potentially allows for treatment effect heterogeneity with respect to unmeasured confounders. This is an important difference compared to ordinary IV methods since they commonly need homogeneous treatment assumption, except for methods under monotonicity assumption (Angrist et al., 1996).

However, the interpretation of the model (D.1) is somewhat difficult since the clustering algorithm is completely nonparametric. When there are some measured covariates related to the unmeasured confounders, the clusters may be interpreted by confirming the measured covariates. For instance, a cluster is consist of many male and over 65 years old, whereas the other cluster is consist of the other subjects. Also, due to the noncollapsibility of the HR in the CPHM, the integrated log hazard ratio between clusters cannot be interpreted simply.

Technically, there are concerns about the identifiability of $\boldsymbol{\beta}_i$ since the baseline hazard λ_{0i} is fully nonparametric. Even if the parameters are identifiable, the variance of the estimators is expected to be larger compared to what was discussed in the main manuscript. Summarizing the above discussions, we recommend modeling the CPHM as (2.2) in the main manuscript for better interpretability of analysis results.

References

Angrist, J. D., G. W. Imbens, and D. B. Rubin (1996). Identification of causal effects using instrumental variables. *Journal of the American Statistical Association 91*(434), 444–455.

Carvalho, C. M., N. G. Polson, and J. G. Scott (2010). The horseshoe estimator for sparse signals. *Biometrika 97*(2), 465–480.

Dahl, D. B., R. Day, and J. W. Tsai (2017). Random partition distribution indexed by pairwise information. *Journal of the American Statistical Association 112*(518), 721–732.

Escobar, M. D. and M. West (1998). Computing nonparametric hierarchical models. In *Practical nonparametric and semiparametric Bayesian statistics*, pp. 1–22. Springer.

Miller, J. W. and M. T. Harrison (2013). A simple example of dirichlet process mixture inconsistency for the number of components. In C. Burges, L. Bottou, M. Welling, Z. Ghahramani, and K. Weinberger (Eds.), *Advances in Neural Information Processing Systems*, Volume 26. Curran Associates, Inc.
